# Uncovering Transcriptional Regulators and Targets of sRNAs Using an Integrative Data-Mining Approach: H-NS-Regulated RseX as a Case Study

**DOI:** 10.3389/fcimb.2021.696533

**Published:** 2021-07-13

**Authors:** Mia K. Mihailovic, Alyssa M. Ekdahl, Angela Chen, Abigail N. Leistra, Bridget Li, Javier González Martínez, Matthew Law, Cindy Ejindu, Éric Massé, Peter L. Freddolino, Lydia M. Contreras

**Affiliations:** ^1^ McKetta Department of Chemical Engineering, University of Texas at Austin, Austin, TX, United States; ^2^ Department of Biochemistry and Functional Genomics, Universitéde Sherbrooke, RNA Group, Sherbrooke, QC, Canada; ^3^ Department of Biological Chemistry and Department of Computational Medicine & Bioinformatics, University of Michigan Medical School, Ann Arbor, MI, United States

**Keywords:** data mining, bioinformatics, bacterial small RNA, regulatory RNA networks, post-transcriptional regulation of gene expression

## Abstract

Bacterial small RNAs (sRNAs) play a vital role in pathogenesis by enabling rapid, efficient networks of gene attenuation during infection. In recent decades, there has been a surge in the number of proposed and biochemically-confirmed sRNAs in both Gram-positive and Gram-negative pathogens. However, limited homology, network complexity, and condition specificity of sRNA has stunted complete characterization of the activity and regulation of these RNA regulators. To streamline the discovery of the expression of sRNAs, and their post-transcriptional activities, we propose an integrative *in vivo* data-mining approach that couples DNA protein occupancy, RNA-seq, and RNA accessibility data with motif identification and target prediction algorithms. We benchmark the approach against a subset of well-characterized *E. coli* sRNAs for which a degree of *in vivo* transcriptional regulation and post-transcriptional activity has been previously reported, finding support for known regulation in a large proportion of this sRNA set. We showcase the abilities of our method to expand understanding of sRNA RseX, a known envelope stress-linked sRNA for which a cellular role has been elusive due to a lack of native expression detection. Using the presented approach, we identify a small set of putative RseX regulators and targets for experimental investigation. These findings have allowed us to confirm native RseX expression under conditions that eliminate H-NS repression as well as uncover a post-transcriptional role of RseX in fimbrial regulation. Beyond RseX, we uncover 163 putative regulatory DNA-binding protein sites, corresponding to regulation of 62 sRNAs, that could lead to new understanding of sRNA transcription regulation. For 32 sRNAs, we also propose a subset of top targets filtered by engagement of regions that exhibit binding site accessibility behavior *in vivo*. We broadly anticipate that the proposed approach will be useful for sRNA-reliant network characterization in bacteria. Such investigations under pathogenesis-relevant environmental conditions will enable us to deduce complex rapid-regulation schemes that support infection.

## Introduction

Bacterial small RNAs (sRNAs) enable rapid post-transcriptional regulatory responses to external stressors that are often present within host environments (Villa et al., 2018), including envelope stress and carbon and metal ion limitation (Holmqvist and Wagner, 2017). Most commonly, these 50-500 nucleotide transcripts (Villa et al., 2018) are induced under distinct environmental conditions and do not encode proteins, with a few exceptions (Gimpel and Brantl, 2017). Instead, sRNAs usually reduce expression of their targets, either via base pairing with mRNAs to occlude the Shine Dalgarno (SD) sequence or recruitment of RNases to degrade mRNAs (Villa et al., 2018; Jørgensen et al., 2020). Less common *trans*-acting sRNA functions enhance mRNA expression by stabilizing mRNAs or activating translation via altering accessibility of the SD (Villa et al., 2018; Jørgensen et al., 2020) or ribosome enhancer regions (Azam and Vanderpool, 2020). Other regulatory consequences of *trans*-acting sRNA-mRNA interactions, such as modulation of Rho-facilitated termination, have also been acknowledged (Bossi et al., 2020). *Trans*-acting sRNAs frequently regulate multiple mRNA targets via imperfect complementarity of binding sites to a cognate mRNA region; this enables multiplicative targeting by a single sRNA and complicates prediction of sRNA-dependent regulatory networks. Additionally, sRNAs can serve as sponges to sequester molecules, including mRNAs (*i.e.*, toxin-antitoxin *cis* sRNA regulation) (Fozo et al., 2008), other sRNAs (Denham, 2020), or regulatory global proteins (Jørgensen et al., 2020). Importantly, the varied mechanisms of sRNA-facilitated regulation are not exclusive [*e.g.*, ArrS targets in *cis* and in *trans* (Melamed et al., 2016), McaS targets in *trans* and sequesters CsrA (Jørgensen et al., 2013)]. The interest in understanding sRNA roles within larger stress-response networks has increased in recent years due to recognized links to pathogenicity (Chakravarty and Massé, 2019) and antibiotic resistance (Mediati et al., 2020).

The past decade has marked a shift from fortuitous sRNA discovery to rational sRNA prediction. Indeed, omics studies, often coupled with unique computational screenings, have enabled identification of numerous sRNAs in both model and non-model bacteria (Leonard et al., 2019; Haning et al., 2020), finding that sRNAs are pervasive in all eubacterial kingdoms (Barquist and Vogel, 2015). In *E. coli* alone, over 85 sRNAs have had their expression biochemically confirmed (Hör et al., 2020). However, condition-specific expression combined with limited sequence conservation among species (Jose et al., 2019) has made rapid detailed sRNA characterization difficult to achieve (Vogel and Sharma, 2005). Even for sRNAs whose native expression has been confirmed, a key question continues to be: how do they enable bacterial survival under stress?

Omics datasets have been crucial for characterizing sRNA target networks by enabling identification of putative sRNA-specific expression and ribosome occupancy effects (Barquist and Vogel, 2015) and of sRNA-enriched binding partners (Carrier et al., 2016). However, given that many sRNAs regulate multiple shared targets simultaneously, there has been a recent focus on method development to offer global resolution. Global *in vivo* methods have been developed to take advantage of the frequent sRNA mechanistic reliance on chaperone RNA Binding Proteins (RBPs), *i.e.*, Hfq (Santiago‐Frangos Andrew, 2018) and ProQ (Holmqvist et al., 2020). For example, crosslinking- and ligation-based methods can uncover unique components of the global sRNA interactome through enrichment via their RBP associations (Hör and Vogel, 2017; Hör et al., 2018; Desgranges et al., 2020). These methods have uncovered thousands of putative regulatory sRNA-mRNA interactions and revealed at least one example of how multiple RBPs (*i.e.*, ProQ and Hfq) co-affect sRNA-sRNA degradation (Melamed et al., 2020).

Although global *in vivo* sRNA profiling methods have provided valuable insights, these methods offer limited resolution for lowly expressed sRNAs that cannot effectively compete for binding to RBPs as well as for sRNAs that do not rely on characterized RBPs for their regulatory activity. This is reflected in the limited characterization of Hfq-independent sRNAs, compared to Hfq-dependent sRNAs, even when regulating shared targets (Guillier et al., 2006). For example, Hfq-dependent sRNA RybB has >15 accepted targets with corresponding molecular interactions characterized (Gogol et al., 2011) while the Hfq-independent phage sRNA IpeX has 1 currently annotated likely-direct target (Castillo-Keller et al., 2006). In light of this challenge, we have developed a global plasmid-based technique to interrogate the regional binding landscape of user-selected sRNAs, independent of RBPs, termed INTERFACE (Mihailovic et al., 2018). Inspired by the ability of *trans*-acting sRNAs to regulate multiple targets with distinct seed regions, this method quantifies the ability of a perfectly complementary antisense RNA (asRNA) to establish basepairs with user-defined 9-16 nt RNA regions *in vivo*; if interaction occurs, this base pairing disrupts a downstream hairpin to enable transcriptional elongation reporter activity. The quantifiable output of this method has previously been coupled to computational predictions (Mann et al., 2017) to enrich for true sRNA targets, identifying mRNA-binding activity in 6 previously uncharacterized sRNAs (Mihailovic et al., 2018).

sRNA-enabled stress response is facilitated by transcriptional regulation that links timing of sRNA expression to dynamic cellular changes. For example, σ^E^-dependent transcriptional activation of MicA and RybB in response to extracytoplasmic stress activates control of their target mRNA networks that support envelope integrity (Gogol et al., 2011). sRNA-regulating DNA binding proteins (DBPs) have been slowly uncovered by ChIP-seq methods that elucidate binding regions and consensus DNA recognition sequences of select DBPs (Hör et al., 2020). However, while some sRNAs have a handful of known regulators, such as MicF and GadF with 8 and 10 reported respectively (Keseler et al., 2011), most sRNAs have few, if any, transcriptional regulators that have been identified. Indeed, less than half of the ~100 annotated sRNAs in *E. coli* have their imparted transcriptional regulation and imparting post-transcriptional regulation characterized (Hör et al., 2020). Furthermore, as roughly 20% of the documented interactions regulating sRNAs involve sigma factors that respond to a multitude of general stresses, it is likely that precise regulation of these individual sRNAs involves tuning by other, more specific regulators (Gottesman, 2019). This underscores the need for standardized global methods that incorporate stress conditions relevant to sRNA expression.

Fortunately, the growing number of publicly-available microarray and omics data stored on multiple databases (Clough and Barrett, 2016) offer a wealth of knowledge on regulatory network logic, reducing the need for exploratory and large-scale experimentation to achieve multiple-condition insights. For example, distinct anticorrelation patterns, observed in a sRNA-centric network inference study considering >40 independent datasets, supported the discovery of reciprocal regulation between sRNA GcvB and the amino acid metabolism transcriptional regulator Lrp (Modi et al., 2011). Recently, the value of integrating global datasets from unique methods to understand regulators has been emphasized (Hör et al., 2018). Indeed, in a recent characterization of the Csr network, an Integrative 4D Omics Approach incorporates multiple unique omics experiments (transcriptomics, proteomics and CLIP-seq) performed in many distinct cell strains and environmental conditions to identify 17 new true targets of the global post-transcriptional regulator CsrA (Sowa et al., 2017). While it is evident that integration of multiple datasets representing many conditions and methods is advantageous for uncovering complex networks, it remains to be widely adopted in a systematic way that investigates the sRNA-ome.

Recently, a global high-resolution *in vivo* protein occupancy display method (IPOD-HR), in which no enrichment for a specific protein is performed, has been shown to capture condition-specific DNA-protein interactions genome-wide (Freddolino et al., 2021). Coupled with motif search, this method offers potential to capture transcriptional regulation by less common factors; to date, this possibility has not been evaluated for sRNAs as IPOD-HR data has only been analyzed in the context of protein coding operons (Freddolino et al., 2021). In this work, we develop an integrative, two-node, datamining approach that utilizes publicly-available omics datasets to understand cellular regulation of and by any sRNA of interest. We name this approach Integrative Datamining for sRNA Regulators ‘n Activity (ID-sRnA). In the transcriptional node of the ID-sRnA approach, IPOD-HR data, coupled with sequence motif searches, is used to suggest DBPs of sRNAs; DBP predictions are then corroborated with available RNA-seq data to assemble a list of high-confidence DBP regulators. In the post-transcriptional node, top-5 high-confidence mRNA targets are compiled from streamlining computational target predictions with regional sRNA accessibility data. We apply this computational approach to 91 annotated *E. coli* K-12 MG1655 sRNAs, showcasing the ability to capture known sRNA regulation and activity. We further propose novel, high-confidence DBP-based regulation of 62 sRNAs and *trans*-targets for 32 sRNAs that are supported by regional accessibility data. In combination, ID-sRnA suggests both putative transcriptional regulation and post-transcriptional activity for 21 sRNAs.

Experimental follow-ups showcase the power of ID-sRnA for the case of RseX, an exemplary enigmatic sRNA originally identified from a computational sRNA screening (Chen et al., 2002) that enhances survival in the absence of cytoplasmic σ^E^ activity via post-transcriptional regulation of outer membrane proteins (OMPs) (Douchin et al., 2006). This survival effect is reminiscent of those corresponding to overexpression of multi-target σ^E^-regulated sRNAs, RybB and MicA (Gogol et al., 2011). However, to date, native RseX expression has not been detected despite numerous independent attempts (Chen et al., 2002; Douchin et al., 2006; Raghavan et al., 2011). Using our integrative analyses, we confirm that RseX transcription is enabled in a strain deleted for the nucleoid-associating protein, H-NS, and validate two novel mRNA targets, *fimB* and *ihfB*, on the basis of their direct interaction with and regulation by RseX *in vitro* and *in vivo*, respectively. Overall we demonstrate the use of integrative methods to elucidate hidden 3-layer regulatory systems, in which DBPs regulate transcription of sRNAs, which, in turn, regulate stability and translation of mRNA targets.

## Materials and Methods

### Selection of sRNA Coordinates for Analysis

The mature sRNA transcript coordinates were defined by RegulonDB (Gama-Castro et al., 2016) for a K-12 MG1655 genome (RefSeq Sequence: NC_000913.3) (**Supplementary Data 1**). For relevant IPOD-HR (Freddolino et al., 2021) data extraction, sRNA coordinates were selected to contain 200 nucleotides (nts) upstream from the nearest RegulonDB transcription start site (TSS) through the mature sRNA transcript region to 10 nts downstream. Therefore, sRNAs that are processed from within or at the 3’ end of longer transcripts (such as 3ETSleuZ, CpxQ, GadF, MicL, nc2, PspH, SroC, SroD, SroE, Tpke11) have the entire upstream transcript included up to the TSS. If no documented TSS existed, the start of the mature transcript region was used as a pseudo-TSS.

### Identification of sRNA-Associated Differential Protein Occupancy From IPOD-HR Data

Previously, z-scaled MG1655 genomic protein occupancy (PO) data IPOD-HR in three distinct conditions (log phase in rich defined medium (RM), stationary phase in RM, and log phase in minimal media) were evaluated by continuous wavelet transform peak calling as implemented in the SciPy python library (Freddolino et al., 2021). Briefly, normalized protein occupancy values across the genome were scanned for maxima in signal above an expected noise threshold. This process was repeated at increasing signal-to-noise (SNR) thresholds of [0.5, 1.0, 1.5, 2.0, 2.5, 3.0, 3.5, 4.0, 4.5, 5.0, 5.5, 6, 7, 8, 9, 10, 12, 15, 20, 25, 30, 40, and 50], resulting in a list of PO peaks across the genome and the maximum SNR value at which each can be detected. Here, these data were reduced to a sRNA-specific subset by identifying peaks that overlap (by at least 5 nucleotides) with genomic regions surrounding sRNA genes (200 upstream of sRNA start to 10 nts downstream of sRNA terminator end for each accepted sRNA sequence). This process was performed on each of three PO datasets, and extracted peaks present in at least one condition were identified. Peaks from different conditions were considered equivalent if they overlapped by at least 50 nts. From this list, each peak was evaluated for differential occupancy defined by (i) peak absence in at least once condition or (ii) SNR ratio ≧ 2 for any condition combination. For each peak corresponding to differential occupancy, the longest possible DNA sequence was exported to a FASTA file.

### Putative DBP Curation and Identification

Probable DBP motifs were identified from genomic sequences with observed differential PO using locally-installed MEME FIMO 4.11.2 (p-value maximum of 1.0e-4) against MEME *E. coli* databases SwissRegulon (Pachkov et al., 2013) and DPInteract (Robison et al., 1998), as well as an *E. coli*-specific database curated in-house from Prodoric2 (Eckweiler et al., 2018). DBPs whose motifs were searched are listed in **Supplementary Data 2**, along with sRNAs in which corresponding motif-harboring differential PO peaks were identified.

### RNA-Seq Curation and Analysis

sRNAs not listed in the K-12 MG1655 genomic GFF (RefSeq Assembly: GCF_000005845.2), were manually inserted using K-12 MG1655 (RefSeq Sequence: NC_000913.3) coordinates of the documented mature transcript defined by RegulonDB (Gama-Castro et al., 2016) (**Supplementary Data 1**).

Appropriate RNA-seq datasets were selected using Gene Expression Omnibus Database (Clough and Barrett, 2016) for *E. coli* K12 MG1655 strains with dataset types limited to expression profiling by high throughput sequencing. Selected datasets contained either DBP deletions or stress conditions of interest. Datasets used with GenBank accession numbers and descriptions are provided in **Supplementary Data 3**.

All datasets were downloaded from the Sequence Read Archive and quality checked by FastQC (http://www.bioinformatics.babraham.ac.uk/projects/fastqc/) prior to further analysis. Using Cutadapt (Martin, 2011), all datasets were quality trimmed for bases with <20 quality scores, and datasets with >15% adapter content had adapters trimmed. Reads were aligned using BWA mem (Li and Durbin, 2010) with alignment quality filter of 30, and gene counts assigned using HT-Seq (Anders et al., 2015) with the above-described genomic GFF. Strandedness was determined based on reported library preparation kits if available. If not available, strandedness was inferred based on “no feature” counts.

Differential expression (DE) analysis was performed in DESeq2 (Love et al., 2014) in R (v. 3.6.3) (https://www.R-project.org/) via LRT test over condition of interest. Statistical significance of sRNA DE was defined as an adjusted p-value < 0.05. To account for DESeq2 bias towards long transcript length, datasets containing fewer than 20 sRNAs differentially expressed were subject to an additional filter to include sRNAs with low base mean counts that would otherwise be excluded from the p-adj filter (base mean count >3 AND p-value < 0.1 AND |Log2FC|>1.5). The log2FC and p-adj values are provided in **Supplementary Data 3**, with the p-adj values that were not reported due to low base mean counts are provided post-analysis through silencing the independent filter in DESeq2. Due to varying degrees of sRNA depth between datasets, statistically significant sRNAs were limited to 20 for each dataset.

DBP motifs corresponding to sequences with differential protein occupancy are listed alongside DE conditions found for each sRNA in **Supplementary Data 2**. Well-known co-factors, activation or repression conditions, and function for each DBP that strongly corresponded with a DE condition tested was noted and compared for each motif and sRNA pair (**Supplementary Data 2**). Of the 102 DBPs searched by FIMO, 48 had at least one corresponding DE condition. High-confidence DBP matches were determined if a DE condition for an sRNA supported the DBP’s known activity.

### Selection of Putative Functional Regions From Accessibility Data

Putative sRNA functional regions were selected from previously-published high-throughput regional RNA accessibility datasets in *E. coli* BW25113 (Mihailovic et al., 2018) (GSE117939) based on activity reminiscent of toehold behavior, namely, drastic accessibility changes between neighboring regions. To capture toehold-like behavior of RNA regions *in vivo*, regional accessibilities from 66 previously-profiled sRNAs shared with the sRNA pool in this work were evaluated for stark accessibility differences between their next-door or overlapping target region neighbors. Specifically, regions with extreme accessibility compared to at least one neighboring region (accessibility difference >0.7 on a normalized 0-1 scale, Student’s 2-tailed t-test p-value < 0.05) were compiled as likely-functional sRNA regions. Importantly, only target regions that were selected based on a machine-learning-based approach were considered; those that were intentionally targeted due to their known binding activity were intentionally excluded from downstream analysis (Mihailovic et al., 2018).

### Computational Target Prediction Filters and Compilation

IntaRNA predictions were collected for all sRNAs investigated in this work against a curated genome-wide mRNA-representative sequence list (Mann et al., 2017). This sequence list was compiled to correspond to annotated mRNA TSS [corresponding to the longest known transcript (Gama-Castro et al., 2016)] to +100, or, for unannotated mRNA TSS, -100 to +100 nts around the start codon was used. Importantly, only the most favorable interaction between a sRNA and each mRNA sequence was considered for downstream processing. Predictions corresponding to each sRNA were subject to exclusion criteria based on thermodynamically-predicted (most favorable) interaction reliance on the likely-functional region, as previously described (Bowman et al., 2020). Briefly, the engagement of at least 80% of the likely-functional region in the predicted interaction was required. Upon exclusion of predicted targets not meeting this threshold, top-5 target predictions were compiled for each likely-functional region (**Supplementary Data 4**) and further flagged if meeting either of two criteria: mRNA function aligned with reported high-confidence sRNA induction conditions (from this study or prior works/documentation, including Gene Ontology annotations) or mRNA identity aligned with sRNA-mRNA pair previously reported from any of 4 independent ligation-based studies (RIL-seq and/or CLASH methods) relying on coimmunoprecipitation of Hfq, ProQ, or RNase E (Melamed et al., 2016; Waters et al., 2017; Iosub et al., 2020; Melamed et al., 2020). For these CLASH and RIL-seq studies, only sRNA-mRNA pairs with FDR < 0.05 and pairs with > 39 chimeras [as identified as a reliable cutoff in (Iosub et al., 2020)] were considered for flagging, respectively.

### Northern Blotting

To measure RseX expression, BW25113, *kanR*-cured BW25113Δ*hns*, and *hns::neo* mutant strains (Yamada et al., 1991), were grown overnight and seeded in LB. Samples were taken at various time points corresponding to distinct growth phases: exponential (4 hours post seeding, OD_600_ ~2.4), transitionary (7 hours, OD_600_ ~3.8), mid stationary (24 hours, OD_600_~3.3), and late stationary (48 hours post seeding, OD_600_ ~3.4). Total RNA was extracted following standard methods (Mihailovic et al., 2018) with slight modifications: 300μL instead of 200 μL of 24:1 chloroform:isoamyl alcohol for separation, 1mL IPA with 1 μL GlycoBlue Coprecipitant (Ambion) instead of the sodium citrate and sodium chloride solution for overnight precipitation, and 95% instead of 75% ethanol/water was used for the first pellet wash. The total RNA was then subjected to previously described northern blot analysis (Haning et al., 2020). In summary, DNA oligonucleotide probes designed complementary to a 5’ region of RseX (**Supplementary Table 1**), as well as the ladder [ΦX174 DNA/HinfI (Promega)], were labeled individually using 20 pmol of olignoucleotide or ladder in a 20 μL kinase reaction consisting of 25 μM [γ-32P]-ATP and 20 units T4 polynucleotide kinase (NEB) at 37°C for 1 hour. Total RNA (~20 μg) for each sample were separated on a 10% denaturing polyacrylamide-urea gel and then transferred to a membrane (Hybond N+, GE Life Sciences) for blotting. Probe hybridization to the membrane was performed using PerfectHyb Plus Hybridization Buffer (Sigma-Aldrich) overnight at 42°C, and then washed three times (first wash: 5 × SSC, 0.1% SDS at 30°C for 20 minutes; second and third wash:1 × SSC, 0.1% SDS at 42°C for 15 min). Membranes were then exposed to a phosphor screen for 72 hours prior to visualization on a Typhoon 9500 (GE). Sizes were estimated by (i) inclusion of an RseX IVT product (known 91 nucleotides) on northern gels and (ii) comparison of band separation of a low-range ssRNA ladder (NEB) to the DNA ladder used in northern gels (ΦX174 DNA/*Hin*fI). RseX quantification using ImageJ (Schneider et al., 2012) was normalized to 5S RNA, which was probed second following the same protocol.

### *In Vitro* Transcription, sRNA Binding and Probing Assays

Binding assays were performed as previously described (Bowman et al., 2020). Briefly, DNA corresponding to RseX and representative mRNA sequences (observed 5’ start from previously published RNA expression data (Sowa et al., 2017) to at least 30 nts downstream of proposed interaction site) were amplified from genomic K-12 MG1655 DNA with an overhanging forward primer to enable *in vitro* transcription (IVT) via T7 MegaSCRIPT kit (Thermo Fisher) (**Supplementary Table 1**). For binding assays, RseX was internally phosphor-labeled by replacing up to 75% of UTP with [α-32P]-UTP in the IVT reaction. All IVT reactions were performed for 6 hours, DNased, then purified via RNA Clean and Concentrator-5 kit (Zymo Research). Non-incorporated labeled nucleotides were removed using Performa DTR gel filtration cartridges (EdgeBio).

For binding assays, 12 uL reactions containing 1.3 pmol internally-labeled RseX and 0-80 (or 0-140 in the case of *ompA*) pmol unlabeled mRNA fragments were suspended in a reaction mixture containing 1X EMSA binding buffer and 10% glycerol. Reactions were denatured at 70°C for 5 minutes and incubated at 37°C for 1.5 hours prior to loading onto a 5% non-denaturing polyacrylamide gel and running at 150V in 0.5X TBE buffer. Phosphor screens were exposed overnight to EMSA gels prior to imaging on a Typhoon 9500.

For probing assays, representative mRNA transcripts were excised from a 7M urea PAGE gel and recovered prior to dephosphorylation via Calf Intestinal Phoshatase (NEB) and 5’-labeling with [γ-32P]-ATP using T4 polynucleotide kinase (NEB). Each sample was then cleaned and concentrated (RNA Clean and Concentrator Kit-5, Zymo Research) prior to lead acetate (PbAc) probing. PbAc probing reactions were performed as previously described (Desnoyers et al., 2009). Briefly, approximately 0.1 μM radiolabeled mRNA transcripts were incubated with or without 1 μM RseX, and reacted with 5mM PbAc at 37°C for 2 minutes. For OH and guanine ladder synthesis, mRNA transcripts were incubated with alkaline buffer (Ambion) for 5 min at 90°C, or RNase T1 (Ambion) for 5min at 37°C. Samples and ladders were loaded on a 0.4mm thickness 10% acrylamide 7M urea sequencing gel and migrated at 38W (OWL S4S Aluminum-backed Sequencing System, Thermo Scientific). Gels were dried for 30 min at 80°C prior to overnight phosphor screen exposure.

### *In Vivo* Reporter Assays

Reporter assays were performed to evaluate regulation of RseX on newly-identified binding partners *fimB* and *ihfB* by quantifying expression of inducible mRNA-representative sequences fused to *gfp* (from a pBTRK derivative) (Youngquist et al., 2013) upon RseX or “empty” induction [from pNM12, (Majdalani et al., 2001)] via flow cytometry. For experimental purposes, the pBTRK plasmid was altered in three ways: (i) replacement of pTrc with pLacO, (ii) replacement of *kanR* gene with *catR* and (iii) shortening of sequence between the multiple cloning sequence and *rrnB1* terminator (relevant oligonucleotides in **Supplementary Table 1**). Representative mRNA sequences for these reporter assays were chosen as the sequences from annotated transcription start sites (fimBp1 for *fimB*) (Gama-Castro et al., 2011) to at least 30 nucleotides downstream of proposed interaction site (**Supplementary Table 1**). These sequences were amplified from the K-12 MG1655 genome and, along with a GFP fragment amplified from pHL1756 (Sowa et al., 2017), inserted into a digested (*Sal*I & *Hin*dII, NEB) pBTRK plasmid derivative via Gibson Assembly. Similarly, the RseX sequence (+26 nucleotides downstream of the transcription stop site, to keep some semblance of the native chromosomal context) were amplified from the K-12 MG1655 genome and inserted into the pBAD-DsrA (Lalaouna et al., 2015) plasmid to replace the DsrA sequence via Gibson Assembly. Corresponding RseX and *fimB* mutations were stringently designed to keep minimum free energy structure consistent (as evaluated via Nupack (Zadeh et al., 2011); < 0.3 kcal/mol deviations tolerated) and, in the case of *fimB*, to additionally maintain similar codon frequencies to the wildtype sequence. RseX mutations were achieved via a Q5 SDM Kit (NEB); the minimal *fimB* mutant sequence was synthetically constructed (IDT) with Gibson Assembly overhangs. All relevant primer and gBlock sequences are supplied in **Supplementary Table 1**.

Plasmids were double-transformed into a *kanR-*cured K-12 MG1655 RseX deletion (Hobbs et al., 2010) in tested combinations. Overnights grown in biological triplicates were seeded 1:100 into six 20 mL LB flasks containing 170 µg/L chloramphenicol and 100 µg/mL carbenicillin. After 1 hr of growth at 37°C and 200 rpm (OD ~0.15), all samples were induced with 1 mM IPTG and 0.05% arabinose. Green fluorescence of ~100,000 cells per sample were measured with BD FACSCalibur and median fluorescence normalized to corresponding empty plasmid controls calculated. Fold changes were statistically compared using Student’s 2-tailed t-test.

## Results

### Development of Integrative Data-Mining Approach to Uncover Regulators and Targets of sRNAs

**Figure 1:**
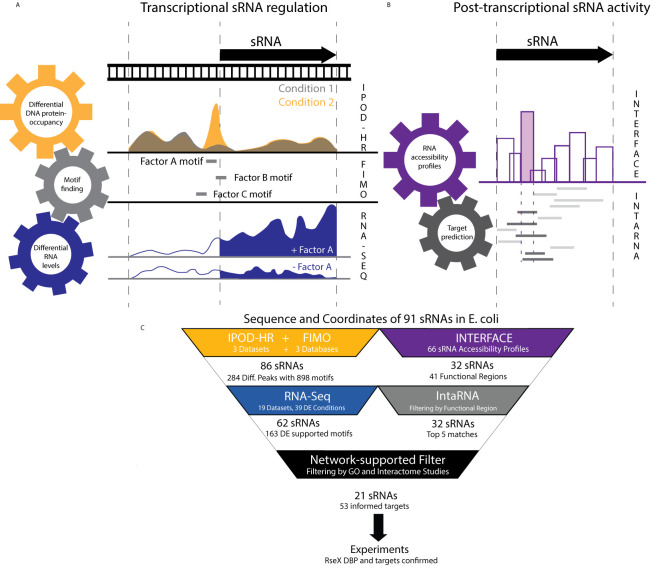
An integrative top-down datamining approach utilizes publicly-available omics datasets to understand cellular regulation of and by any sRNA of interest. **(A)** The ID-sRnA approach is split into two distinct nodes. In the transcriptional regulation characterization node, DNA sequences corresponding to relevant sRNA transcription sequence space [-200 to +10] are narrowed to those that exhibit condition-specific occupancy. For selected 60-185 nt genomic fragments, DBP motif searching is performed to compile a set of putative regulators. High-confidence regulators are selected as DBPs for which differential RNA expression corroborates putative DBP binding. **(B)** The post-transcriptional sRNA characterization node of ID-sRnA relies on coupling of high-throughput regional accessibility data with computational target predictions. Region 3 (shaded) is selected as a likely functional region due to its toehold-like activity; namely, high accessibility with surrounding low accessibility. Target predictions are flagged by reliance of the lowest energy sRNA-mRNA interaction on the proposed sRNA functional region (dark) and re-ranked to exclude those that do not rely on the functional region for interaction. **(C)** Specific number of sRNAs and datasets used for each step of the ID-sRnA pipeline are highlighted, quantifying the amount of filtering performed at each step. 91 sRNAs were considered in which 62 and 32 sRNAs remain in the transcription and post-transcriptional regulation nodes, respectively. Fifty-three accessibility-informed targets corresponding to 21 sRNAs are supported by sRNA regulation factors identified through node 1 and/or previously documented sRNA characterization. Results for RseX are followed-up experimentally to confirm a negative DBP regulator (H-NS) as well as two novel targets, *fimB* and *ihfB*.

We have developed a computational approach (ID-sRnA) for identifying experimentally-supported regulators and targets of bacterial sRNAs by coupling multiple large and distinct omics datasets as well as bioinformatic prediction tools. The ID-sRnA approach is split into two distinct characterization nodes– for transcriptional (**Figure 1A**) and post-transcriptional regulation (**Figure 1B**). The outputs of these two nodes, respectively, are (i) identities and putative binding positions of IPOD-HR- and Next Generation Sequencing (NGS)-supported DBPs, namely, transcription factors (TFs), sigma factors, or nucleoid-associating proteins (NAPs), that may influence sRNA-specific expression, and (ii) computational sRNA target predictions informed by sRNA regional hybridization patterns *in vivo*, many of which are further supported by Gene Ontology analysis. In this work, the ID-sRnA approach is exemplified for 91 annotated sRNAs in *E. coli* K-12 MG1655 (**Supplementary Data 1**); however, the approach can be applied to any bacterium of interest pending data availability. Notably, all sRNAs investigated in this work have homologs in the pathogenic EHEC O157:H7 strain (**Supplementary Data 1**).

To uncover putative native transcriptional regulation of sRNAs, the transcriptional node corroborates conditional global DNA protein occupancies (POs) with corresponding RNA expression in a stepwise filtering process. First, for 91 biochemically-confirmed sRNA sequences (**Supplementary Data 1**), we performed a search of condition-specific PO on genomic positions from -200 to +10 of the corresponding encoding DNA (a wide span to include potential NAPs) within a publicly-available IPOD-HR dataset (Freddolino et al., 2021) under three distinct growth conditions (rich media log phase, minimal media log phase, rich media stationary phase). Importantly, this method captures genome-wide PO independent of traditional immunoprecipitation (Freddolino et al., 2021). By normalizing to RNA Polymerase-derived occupancies, these datasets encompass protection by any DBP. IPOD-HR has previously been successful in capturing known condition-specific TF binding, including nutrient-dependent ArgR at the *argA* promoter under minimal media conditions (Freddolino et al., 2021). To capture the environmental-responsive nature of sRNAs that is critical to pathogenesis (Chakravarty and Massé, 2019), the first step of the ID-sRnA approach was the selection of 284 DNA PO peaks based on their condition-specific occupancy behavior (signal-to-noise ratio, SNR, ≥ 2 between 2 conditions, or no appreciable peak in at least 1 condition). To identify putative DBPs corresponding to these 284 sequences, motif scanning was performed against 3 curated *E. coli* databases representing 102 unique DBPs (see Methods) using FIMO 4.11.2 (Grant et al., 2011) with user-specified constraints (p-value < 1e-04).

The final step (step 3) of the transcriptional node consults publicly available RNA-seq data to lend support or opposition to putative DBP-based transcriptional regulation as inferred from steps 1 and 2. For this step, we mined 19 experimental RNA expression datasets, representing 15 unique DBP deletion strains as well as numerous host infection-mimicking conditions including nutrient limitation, metal ion limitation, and low pH (listed in **Supplementary Data 3**). Importantly, this collected set of RNA expression profiles contains well-known activation or repression conditions corresponding to 48 (of 102 total) DBPs with associated motifs that were utilized for scanning in step 2 (**Supplementary Data 2**). Using DEseq2 (Love et al., 2014), sRNAs with differential expression were compiled for each analyzed condition (see Methods). This analysis narrowed the list of putative sRNA-regulating DBPs down to 163 (18% of those suggested by coupled PO analysis and motif search alone), hereafter referred to as our high-confidence pool (**Figure 1C**) (bolded in **Supplementary Data 2**). A few of these promising DBP-sRNA pairs are further detailed in the discussion. Interestingly, 30% of high-confidence putative sRNA regulators involve TF or sigma factor motifs downstream of the transcription start site, which may suggest important regulation outside of RNAP recruitment to or occlusion of the promoter region (see Discussion).

In the post-transcriptional node of the ID-sRnA pipeline, we integrate omics-enabled regional (9-16 nt) RNA accessibility data, previously collected for a large pool of *E. coli* sRNAs, (Mihailovic et al., 2018) with computational target predictions to compile an accessibility-informed, filtered set of top-5 (arbitrary cutoff for rank comparison purposes) putative *trans* targets. The method of informing computational predictions with *in vivo* information, *i.e.*, extremely accessible sRNA regions, was previously shown to increase positive predictive value for a subset of *E. coli* sRNA targets (Mihailovic et al., 2018; Bowman et al., 2020). It is important to note that our selection of likely functional regions in this approach differs from previous efforts; as opposed to focusing on extreme accessibility, we instead exploit the unique characteristic of high regional accessibility with neighboring low accessibility which appeared suggestive of seeding or toehold regions in sRNAs DsrA and RprA (Mihailovic et al., 2018). Specifically, for 66 sRNAs with clear accessibility profiles in wildtype BW25113 (Mihailovic et al., 2018), putative regulatory sites were selected as sRNA regions with high accessibility delta (difference > 0.7 on a normalized scale, 2-tailed t-test p-value < 0.05) compared to at least 1 nearest neighboring regions (**Figure 1B**). Using this selection criterion and further excluding antisense sRNAs that regulate toxic proteins, we identified 41 regions within 32 sRNAs as likely functional sites (**Supplementary Data 1**). For sRNAs with known Hfq dependencies, reduced accessibility of 5 putative regulatory sites in a *kanR*-cured isogenic Δ*hfq* strain (Methods) lent further confidence to region selection (**Supplementary Data 1**) given the accepted role of Hfq in rearranging sRNA structure for optimal target base-pairing (Santiago‐Frangos Andrew, 2018) that has previously been captured by regional accessibility studies (Mihailovic et al., 2018).

These 41 sites were used as filters for IntaRNA predictions to rank all predictions from highest to lowest likelihood of being true sRNA targets, as previously described (Bowman et al., 2020). Briefly, predicted target mRNAs were reranked under the constraint that 80% of the proposed sRNA seed region was involved in predicted mRNA binding. Upon combing of predictions, top-5 putative targets for each of the 41 sites were compiled for a total of 201 targets. In light of regulatory sRNA activity commonly being tailored to corresponding induction conditions (Gottesman, 2019), we flagged top-5 predicted mRNA targets with documented protein function that aligns with sRNA expression regulation (as inferred by previously documented regulators and/or high-confidence regulators from node 1) or Gene Ontology. This analysis supports a quarter of the compiled high-confidence putative targets (**Supplementary Data 4**). We additionally flagged top-5 predicted mRNA targets that are reinforced by previously-published large scale *in vivo* interactome data, finding that approximately 5% of putative sRNA-target interactions have been observed in these large studies (Melamed et al., 2016; Waters et al., 2017; Iosub et al., 2020; Melamed et al., 2020). This low proportion can be partially attributed to the complete lack of representation in interactome studies for 9 of 32 considered sRNAs (**Supplementary Data 4**) (Melamed et al., 2016; Waters et al., 2017; Iosub et al., 2020; Melamed et al., 2020), likely due to low abundance or RBP independence.

**Figure 2:**
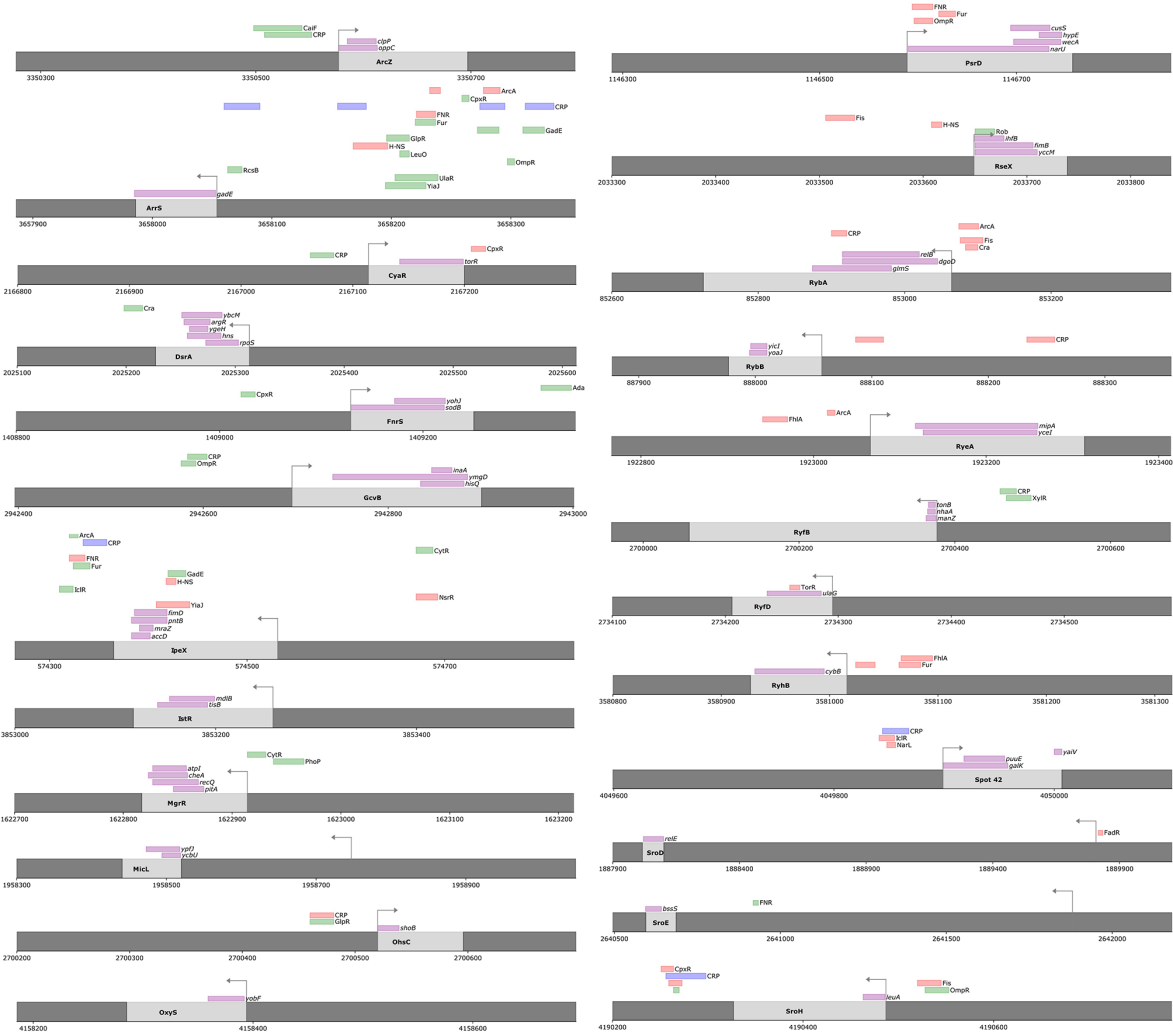
High-confidence regulators and/or targets for 24 sRNAs are suggested by the ID-sRnA approach. Upon using the ID-sRnA approach for a set of 91 *E. coli* sRNAs, both transcriptional and post-transcriptional regulation are proposed for 21 sRNAs and post-transcriptional regulation only is suggested for 3. Genomic context of each sRNA is shown, with (i) marked position ranges corresponding to identified motifs for putative high-confidence sRNA-regulating DBPs (repressors as red, activators as green, and dual-functions as blue) (ii) marked ranges corresponding to thermodynamically-predicted binding positions for putative high-confidence mRNA targets (magenta). Arrows indicate position of the TSS.

**Figure 3:**
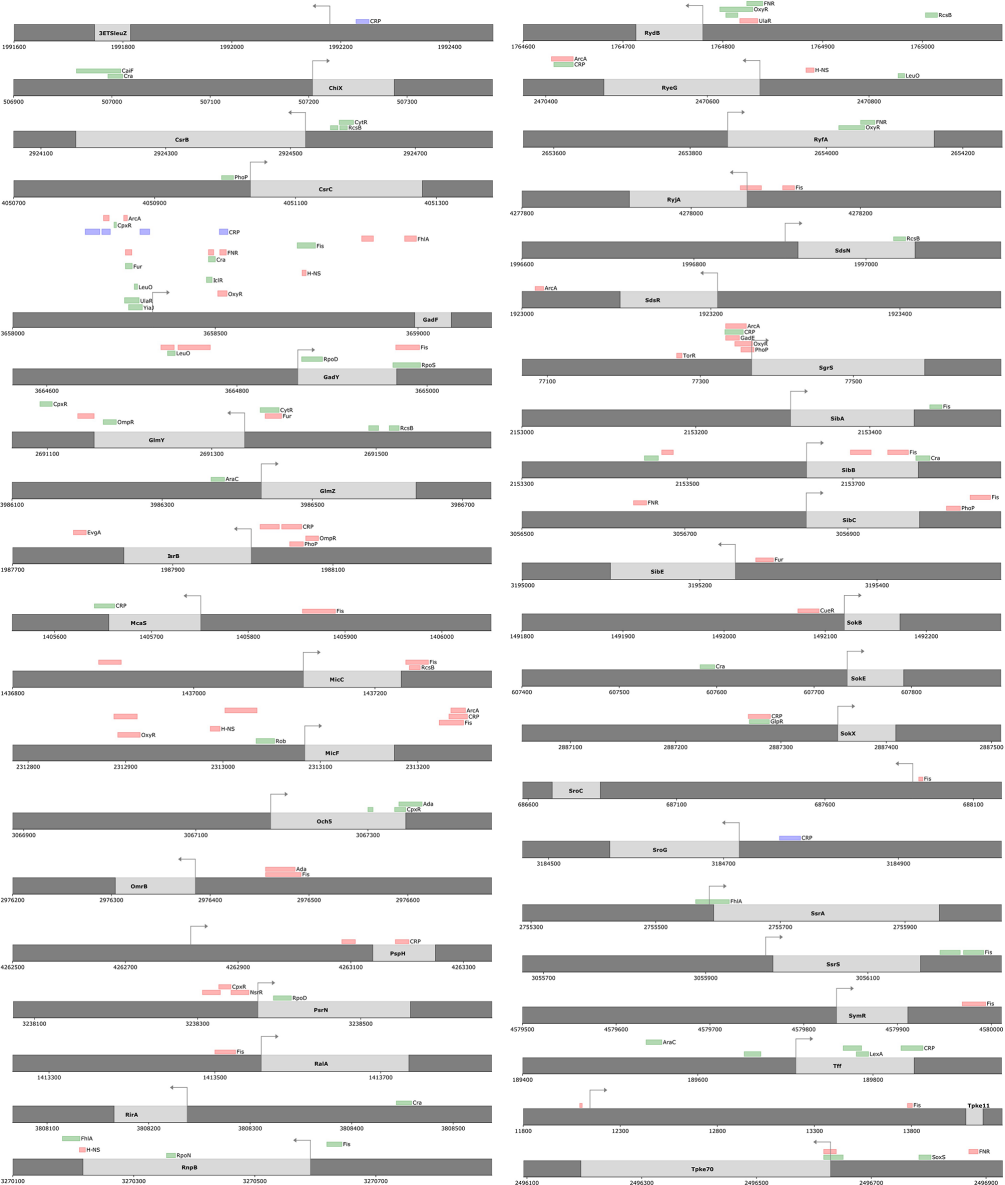
High-confidence regulators only are proposed for 41 sRNAs using the ID-sRnA approach. Transcriptional regulation only is proposed for 41 sRNAs that did not have a seed region identified from the post-transcriptional ID-sRnA node. Genomic context of each sRNA is shown, with (i) marked position ranges corresponding to identified motifs for putative high-confidence sRNA-regulating DBPs, as in **Figure 2** (repressors as red, activators as green, and dual-functions as blue). Arrows indicate position of the TSS.

The proposed ID-sRnA approach integrates data from multiple independent high-throughput studies to propose sRNA regulators and targets with high confidence. In total, for 91 annotated *E. coli* sRNAs, high-confidence regulators and/or targets are listed for 65— 21 sRNAs with both transcriptional and post-transcriptional regulation (**Figure 2**), 3 with only post-transcriptional regulation (**Figure 2**), and 41 for which only transcriptional regulation is suggested (**Figure 3**). We investigate these results in detail for signatures of expected regulation as well as novel regulation in the following sections.

### Known Transcriptional sRNA Regulation Captured by Integrative Pipeline

**Figure 4:**
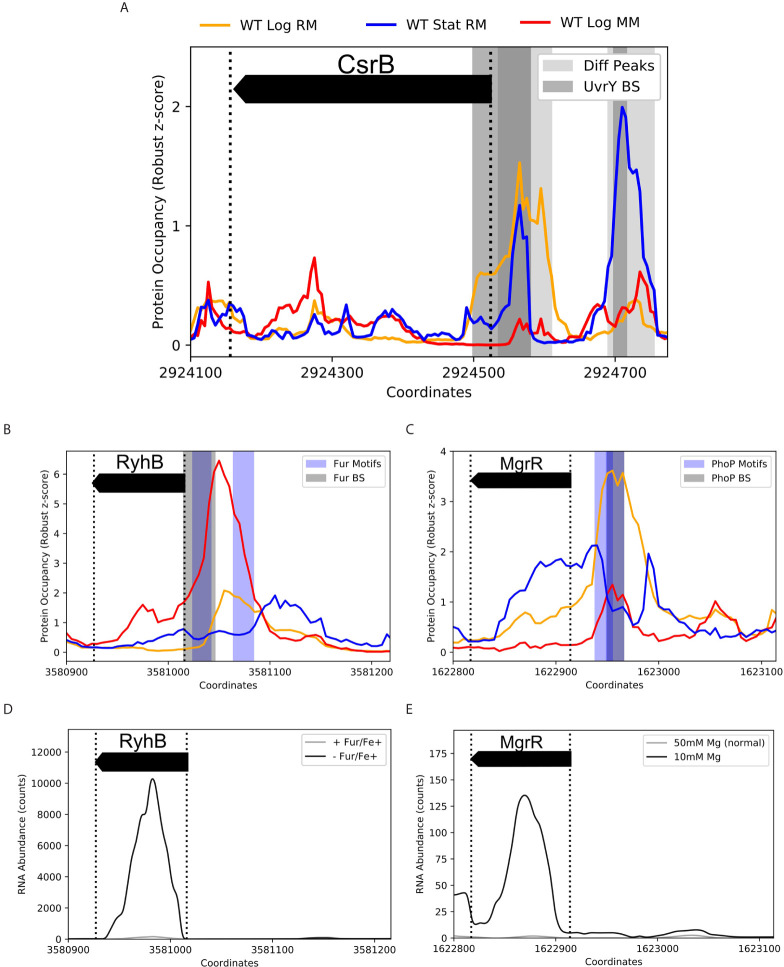
Coupling IPOD-HR with FIMO captures known condition-specific protein occupancy upstream of sRNA promoter regions. Protein occupancies (PO) corresponding to 3 environmental conditions are displayed by line color (top) for K-12 samples collected in log or stationary phase grown in rich or minimal media (RM, MM). Shaded regions correspond to differential PO peaks (light grey), known binding sites (BS, grey), or FIMO-identified motifs (Motifs, blue), as listed. **(A)** Two differential PO peaks within -250 to +10 (with respect to TSS) of sRNA CsrB were identified. These regions overlap two previously-identified binding sites of UvrY (DNase I Footprinting: -192 to -174, ChIP-exo: -222 to -142 (not shown) and -56 to +25) (Zere et al., 2015). The binding of UvrY is known to activate CsrB transcription, however, coordination between sites is not well understood. Interestingly, the two peaks differ in presence between log and stationary phase, and between RM and MM, suggesting varying UvrY modes of binding. A UvrY consensus sequence was not in any of the tested motif databases, and therefore could not be captured by the FIMO search. B/C. PO of approximately [-200 to +10] nucleotides of the sRNAs RyhB **(B)** and MgrR **(C)** with documented BS and FIMO captured motifs of the iron-responsive Fur regulator [EMSA: -30 to +1 (Chen et al., 2007)] and the cation-responsive PhoP regulator [consensus motif identification: -52 to -36 (Moon and Gottesman, 2009)], respectively. **(D)** RNA-seq comparison between a *fur* deletion and wildtype in the presence of iron (Seo et al., 2014) supports the role of Fur repressing RyhB transcription, enabling Fur-RyhB to be captured as a high-confidence DBP-sRNA pair by the data-mining approach. **(E) ** MgrR is highly expressed under magnesium deprivation (McClune et al., 2019). As PhoP is known to activate transcription in response to magnesium deprivation, among other divalent cation limitations, it is likely that IPOD-HR data captured calcium-dependent differential PhoP occupancy between RM (4µM CaCl_2_) and MM (400µM CaCl_2_) **(C)**.

To validate the effectiveness of ID-sRnA in capturing sRNA regulation, we benchmarked the transcriptional node of the integrative pipeline against 39 sRNAs that have documented DBP-facilitated transcriptional regulation (Keseler et al., 2011; Hör et al., 2020). Importantly, IPOD-HR data alone is able to capture PO expected of known sRNA regulators in the tested conditions. For example, two PO peaks upstream of the CsrB sRNA that are present during stationary phase (-222 to -142 and -56 to 25) align well with *in vivo*-determined UvrY binding sites (Zere et al., 2015) (**Figure 4A**). Notably, the downstream PO peak (-56 to +25) persists during log growth although the upstream occupancy is severely diminished. These results support prior *in vitro* footprinting that identified only the upstream binding site (-192 to -174) (Zere et al., 2015), suggesting that other factors facilitate UvrY binding at the promoter region in a nutrient-specific manner. Although the IPOD-HR support of UvrY-CsrB regulation is favorable, this is one example of many potential DBP-sRNA pairs that will not be flagged as “high-confidence” due to the lack of a known consensus motif of the associated DBP (UvrY) that limits identification in the motif-search step of the pipeline. This observation emphasizes the conservative nature of our stepwise method, as well as highlights that many of putative sRNA regulators outside of the high-confidence pool (**Supplementary Data 2**) may merit experimental follow-up.

For many DBPs that have documented consensus motifs, corresponding known sRNA regulation was successfully identified via motif search within condition-specific PO peaks (~40%, **Supplementary Data 2**). In **Figures 4B–E**, we showcase the ability of the transcriptional node to capture RyhB and MgrR regulation by tailored metal-specific factors (Fur, Fe^2+^-regulated, and PhoP, Mg^2+^-regulated, respectively). Importantly, these two sRNAs have been recently shown to directly post-transcriptionally regulate expression of the locus of enterocyte effacement in EPEC (Bhatt et al., 2017), emphasizing the importance of metal ion response networks in pathogenicity. Using FIMO, an expected Fur motif (p-value < 1e-06) was identified within strong, differential PO peaks in the RyhB promoter region during log growth in minimal media (SNR>10); an additional identified Fur motif (p-value < 1e-04) aligns with the smaller PO peak in log growth in rich media (SNR>4.5) (**Figure 4B**). Although iron ion (Fe^2+^) concentrations between these two medias are equivalent, it is likely that iron uptake rates vary considerably due to altered nutrient availability, contributing to the dynamic PO peaks. Similarly, expected PhoP regulation is accurately suggested from motif search of condition-specific peaks upstream of the MgrR transcription start site (TSS) (**Figure 4C**) (Moon and Gottesman, 2009). Importantly, the PO peak corresponding to the PhoP motif identified is nonexistent in rich media-based stationary growth, and grows in magnitude and width between minimal and rich media-based log growth. This seemingly reflects the activation of PhoP regulator PhoQ under Ca^2+^ deficiency (4µM in rich media *vs* 400 µM CaCl_2_ in minimal media), a documented response that is appreciable although less efficient than response to Mg^2+^ deficiency (Véscovi et al., 1997).

Many of these proposed DBP-sRNA pairs are further supported by differential RNA expression behavior, binning them into a high-confidence sRNA regulator pool (163 total, corresponding to 62 sRNAs). Twelve sRNAs (of 39 sRNAs with known regulators) have at least one previously-documented DBP association represented within this list (**Table 1** and **Supplementary Figure 1**), including RyhB and MgrR. For example, differential RyhB expression is observed under Fur knockout, supporting RyhB as a target within the Fur regulon (Seo et al., 2014) (**Figure 4D**). In contrast, the PhoP-MgrR regulon is one of many DBP-sRNA pairs that can be categorized as high confidence despite DBP-specific RNA-seq (*i.e.*, +/- PhoP) being unavailable; rather, a stress known to induce expression of PhoP is considered (divalent cation depletion). In this way, MgrR regulation by PhoP is further corroborated by differential expression of MgrR observed between 10 mM and 50 mM Mg^2+^ (McClune et al., 2019) (**Figure 4E**).

**Table 1:** Documented DBP-sRNA regulons are captured by node 1 of the ID-sRnA approach.

sRNA	sRNA Function	DBP	DBP Function	Suggested DBP Effect	DESeq Evidence	Reference of DBP Binding Site
ArrS	Stabilizes *gadE* maturation, Regulates acid response	**GadE**	Regulates acid response	Activator	–Δ*gadE*	Ma, *J Bacteriol* (2004)
CyaR	Represses porin synthesis and group behavior	**Crp**	Catabolism of secondary carbon sources	Activator	–Δ*crp*/Glucose, –Δ*crp*/Glycerol	Johnson, *J Mol Biol* (2008)
	Represses porin synthesis and group behavior	**CpxR**	Regulates envelope stress response	Repressor	-H2O2	Vogt, *J Bacteriol* (2014)
GadF	unknown, generated from 3’ UTR of *gadE*	Crp	Catabolism of secondary carbon sources	Repressor, Dual	++Δ*crp*/glucose, ++Δ*crp*/fructose, ++Δ*crp*/Glycerol, ++Acetate	Hirakawa, *J Bacteriol* (2006)
		ArcA	Regulates anaerobic metabolism	Activator	+Anaerobic	Deng, *Front Microbiol* (2013)
		H-NS	Nucleoid-Associated	Repressor	++Δ*hns*	Krin, *BMC Microbiol* (2010)
GadY	Activates acid response	RpoS	Stationary phase regulator	Activator	–Δ*rpoS*, +MidStat	Opdyke, *J Bacteriol* (2004)
IsrB	Contains peptide coding sequence	**Crp**	Catabolism of secondary carbon sources	Repressor	–Fructose, –Acetate	Hemm, *J Bacteriol* (2010)
McaS	Regulates flagellar motility and biofilm formation	**Crp**	Catabolism of secondary carbon sources	Activator	–Δ*crp*/Glucose, –Δ*crp*/Glycerol	Thomason, *Mol Microbiol* (2012)
MgrR	Represses modified lipopolysaccharide	**PhoP**	PhoPQ two-component system	Activator	++Low Mg	Moon, *Mol Microbiol* (2009)
RnpB	Subunit of ribonuclease	**Fis**	Nucleoid-Associated	Activator	–Δ*fis*	Choi, *Mol Cells* (2005)
RyhB	Represses production of iron-containing proteins	**Fur**	Iron regulator	Repressor	++Δ*fur*/Fe+, -Anaerobic	Chen, *NAR* (2007)
Spot42	Represses galactokinase degradation	**Crp**	Catabolism of secondary carbon sources	Activator, Dual	–Δ*crp*/Glucose, –Acetate, –Fructose, –Glycerol	Polayes, *J Bacteriol* (1988)
SroD	Unknown, generated from 3’ UTR	FadR	Fatty acid regulator	Repressor	++Δ*fadR*	Feng, *PLoS One* (2012)
SsrS	Represses polymerase and σ^70^ activity	**Fis**	Nucleod Associated	Activator	–Δ*fis*	Neusser, *Biol Chem* (2008)

Of 39 sRNAs with well-characterized transcriptional regulators, 12 were captured as high-confidence DBPs by the integrative approach. Bold factors are documented in a recent sRNA review (Hör et al., 2020). Characters (+/-) indicate log2 fold change strength with double characters (++/–) signifying greater/less than |1.5|.

Altogether, these results support the identification of true sRNA-regulating DBPs from the transcriptional node of the ID-sRnA approach. It is interesting to note that consideration of a few, nutrient-tailored IPOD-HR conditions enabled the pointed capture of sRNA-regulating DBPs whose accepted inducing stresses are not necessarily represented within these data (*e.g.*, acid stress of GadE-ArrS). In addition to showcasing the power of this approach to identify a novel regulator for sRNA RseX in the results below, we examine other promising predicted regulator-sRNA pairs in the Discussion.

### Known Post-Transcriptional sRNA Regulation Captured by Integrative Pipeline

To benchmark sRNA post-transcriptional regulation suggested by ID-sRnA, namely, the restriction of target predictions to sRNA regions previously observed to exhibit toehold-like accessibility behavior, we considered 12 characterized sRNAs from the pool of 32 with accessibility-identified functional regions. These *trans*-acting sRNAs were selected on the basis of *in vivo*-characterized target regulation (ArcZ, CyaR, DsrA, FnrS, GcvB, IstR, MicL, MgrR, OxyS, RybB, RyhB, Spot42) (Mihailovic et al., 2018). Within this subset, our proposed functional regions overlaps known mRNA-binding coordinates of nine of these sRNAs (ArcZ, DsrA, FnrS, GcvB, IstR, MgrR, RybB, RyhB, Spot42) (**Supplementary Data 1**). Upon filtering corresponding target predictions by position coordinates outlined in **Supplementary Data 1**, known targets are captured in the filtered top-5 high-confidence targets for 6 sRNAs: DsrA, FnrS, GcvB, MgrR, and Spot42 (**Supplementary Data 4**). Notably, true targets corresponding to half of these sRNAs would not have been captured within the top-5 without the functional region filter (FnrS:*sodB*, GcvB:*inaA*, MgrR:*pitA*). It is interesting to note that the phenotypic relevance of true targets captured for each of the referenced examples aligns well with accepted stress-specific expression of the sRNA although the INTERFACE assay was performed in non-stress (*i.e.*, non-enriched) conditions. However, some true sRNA-target pairs were not captured in top-5 by our approach, despite correctly assigning a region overlapping a known regulatory site as likely functional (*e.g.*, ArcZ). This may be attributed to low thermodynamic ranking of known targets (#4014 for known target *eptB*) from inaccurate reflection of true *in vivo* mechanisms.

These observations may additionally harbor worthwhile molecular insights for even previously-documented post-transcriptional sRNA regulation. For example, the post-transcriptional node of the ID-sRnA pipeline successfully captured known MgrR target *pitA*, encoding for an inorganic phosphate transporter, but not other known targets [*e.g.*, *eptB, soxS, ygdQ* (Hör et al., 2020)]. Indeed, all targets, including *pitA*, are known to bind within an extremely low-accessibility MgrR region (region 7, **Supplementary Figure 2**); however, *pitA* is the only target with predicted binding extending through the extremely accessible likely-functional region (region 6, **Supplementary Figure 2**) (Yin et al., 2019). It will be interesting to identify whether this extended interaction range allows for competitive displacement of other MgrR targets by *pitA*.

Overall, these results highlight the utility of combining multiple datasets to suggest high-confidence *in vivo* regulation of sRNAs. Furthermore, selected putative functional regions that do not corroborate with corresponding documented regulatory sites at all may point to undiscovered binding sites or unrecognized sRNA regulatory activity, as we later discuss.

### Integrative Approach Uncovers H-NS as Negative Regulator of sRNA RseX

To challenge the ID-sRnA pipeline, we investigated an sRNA with limited accepted stress-survival post-transcriptional activity, and elusive native expression conditions. RseX, RNA suppressor of extracytoplasmic stress protease, was initially identified as a suppressor of RseP deletion toxicity from a plasmid-based screening. RseP is one of two mandatory regulators responsible for activating the σ^E^-mediated response by relieving it from membrane sequestration upon extracytoplasmic stress. The role of RseX in cellular survival under the toxic RseP deletion has been attributed to post-transcriptional, Hfq-dependent repression of *ompA* and *ompC* (Douchin et al., 2006). The ability of an sRNA to compensate for the widespread transcriptional and post-transcriptional envelope homeostasis regulation of σ^E^ raises questions concerning: under which cellular conditions is such complementation advantageous? In other words, when is RseX natively expressed?

Previously, 5’ end mapping on a strain containing a plasmid from which RseX suppressor activity was originally identified suggested that RseX is produced as a primary transcript (Douchin et al., 2006). This is in agreement with an upstream σ^70^ consensus sequence and Rho-independent terminator that enabled its identification as a putative sRNA almost two decades ago (Chen et al., 2002). However, the role of RseX as an extracytoplasmic stress-responsive regulator remains elusive due to (i) lack of a consensus binding sequence for any extracytoplasmic stress-associated DBP (*e.g.*, σ^E^, OmpR) and (ii) undetectable native expression.

**Figure 5:**
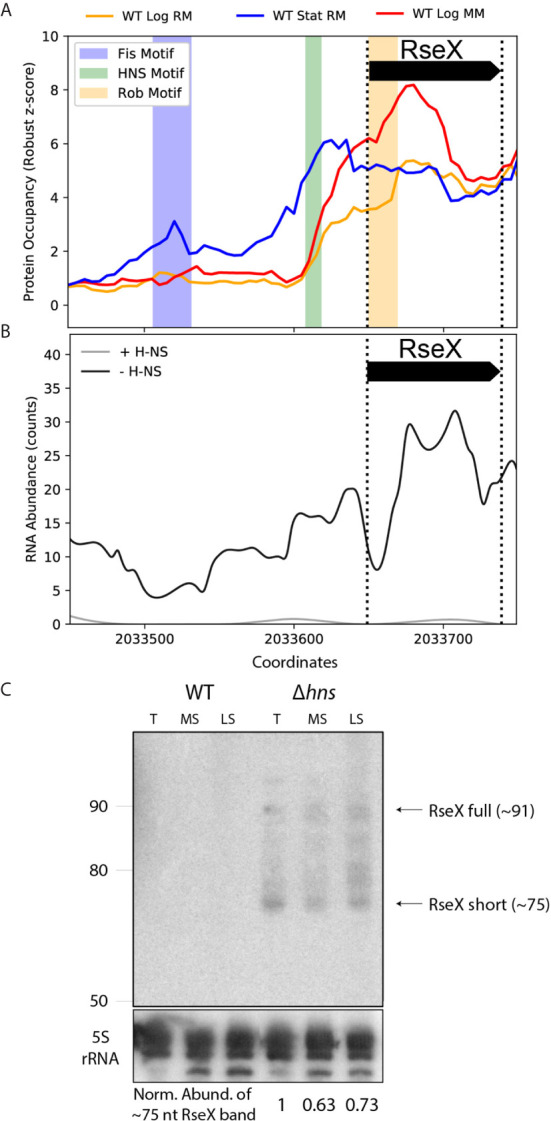
Protein occupancy data and native expression probing support transcriptional RseX repression by nucleoid-structuring protein, H-NS. **(A)** PO data for the approximate [-200, +10] accepted RseX genomic region (bounded by dashed vertical lines). Three significant PO peaks are observed that contain motifs corresponding to Fis (p-value of 6.1e-05), H-NS (p-value of 5.8e-05), and Rob (p-value of 5.2e-05). **(B)** RNA-seq counts corresponding to pooled strains of H-NS knockouts *versus* pooled strains with no modifications to genomic *hns* (Srinivasan et al., 2013). Expression of RseX and surrounding areas is enhanced in the absence of H-NS. **(C)** Northern blotting for RseX in wildtype BW25113, and an isogenic, cured *hns*-deletion strain, grown in LB, at exponential transition to stationary (T, 7 hours post seeding), mid stationary (MS, 24 hours post seeding) and late stationary (LS, 48 hours) growth phases. Lanes for the different cell strains are indicated. RseX expression (documented sRNA, ~91nt) is seen in the Δ*hns* strain at all sampled timepoints besides exponential phase (not shown). A smaller band corresponding to RseX, “RseX short” (~75 nt, size estimated from ladder interpretation, left, as described in Methods), suggests post-transcriptional processing or early transcription termination. RseX expression is not observed in a wildtype strain at any growth phase. Expression of the ~75 nt RseX short transcript is normalized to a 5S rRNA control (bottom).

We first tested the ability of ID-sRnA to uncover RseX regulators that would indicate cellular conditions under which RseX is natively produced. The ID-sRnA transcriptional node proposes three putative RseX regulators as high-confidence (**Figure 5A** and **Supplementary Data 2**): Fis, corresponding to an upstream stationary phase-specific peak at -143 to -118 (p-value = 6.08e-05), H-NS, corresponding to the stationary phase-specific peak at -42 to -32 (p-value = 4.7e-05), and Rob, corresponding to log phase-specific peak at +1 to +20 (p-value = 5.2e-05). Given the proximity between the RseX promoter and the identified motif corresponding to the H-NS global silencer, we suspected that H-NS was in part responsible for the lack of native RseX detection. It should be noted that H-NS is a nucleoid-associating protein (NAP) that acts via chromatin structure remodeling at curved, often AT-rich sites (Fang and Rimsky, 2008). Importantly, the hypothesized role of H-NS in RseX regulation was strongly supported by RNA-seq datasets in relevant genomic deletions (**Figure 5B**) (Srinivasan et al., 2013). Specifically, significantly increased expression of RseX was identified between pooled samples of *hns* deletions (Δ*hns*, Δ*hns/stpA*, Δ*hns/hha*, Δ*hns/ygdT*) compared to a wildtype strain (log2FC = 5.77 and padj = 7.24E-03); interestingly, the observed increase in RseX expression was heightened in the *hns*/*stpA* double-deletion when analyzed alone with respect to wildtype (log2FC = 7.23, padj = 4.26E-07) compared to insignificant differential expression in the single H-NS mutant alone (log2FC = 3.39 and padj = 0.43). This behavior is indicative of epistatic H-NS regulation, in which non-essential gene repression by H-NS is “backed up” by StpA [a phenomenon oftentimes seen for horizontally acquired genes (Srinivasan et al., 2013)], aligning with possible *Salmonella* origins of RseX given its genomic proximity to *yedS* (Douchin et al., 2006). Importantly, the magnitude and significance of transcriptional upregulation in pooled Δ*hns* strains was distinct for RseX compared to other sRNAs implicated in OMP regulation (σ^E^-activated RybB, MicA and EnvZ/OmpR- activated MicF, OmrA/B, MicC) (**Supplementary Figure 3**). These expression patterns suggested unique H-NS-mediated transcriptional regulation of RseX compared to other shared-target regulators.

To validate transcriptional insights predicted by the ID-sRnA approach, native RseX northern blotting was performed using a radiolabeled oligonucleotide targeting near the 5’end of the transcript (nts +12 to +38 from 5’ of longest 5’ RACE-detected sequence) (Douchin et al., 2006) within total RNA extracted from BW25113 and an isogenic Δ*hns* strain (Baba et al., 2006). RNA samples were collected at multiple growth phases—exponential (not shown), transitionary, mid stationary, and late stationary (**Figure 5C**). In accordance with previous efforts to detect RseX expression (Chen et al., 2002), no transcript was observed in total RNA extracted from wildtype cells, regardless of growth phase. In contrast, RseX-specific expression corresponding to the expected size (~91 nts) was detected under H-NS deficiency in most growth phases (with the exception of exponential phase, not shown), suggesting that RseX transcription is negatively regulated by this histone-like NAP. Lack of observable RseX expression in a Δ*hns* strain during exponential growth may be an effect of compensatory silencing by the H-NS partner protein StpA, whose expression is predominantly limited to the exponential growth phase (Ali Azam et al., 1999).

It is also important to note that for all conditions under which RseX expression was detected, smaller overlapping transcript products were also observed, with a prominent band at approximately 75 nucleotides (size estimated as described in Methods). In light of experimental 5’ RACE support of a putative σ^70^ RseX promoter (Douchin et al., 2006), it is possible that the shorter transcripts detected are due to 3’ end processing or early termination. Distinct bands corresponding to the ~75 nucleotide RseX product were also observed in all growth phases (including exponential) in an H-NS-mut strain, *hns::neo* (Yamada et al., 1991) (**Supplementary Figure 4**), which genetically encodes for only the last 37 amino acids. This mutation strain is believed to support some dimerization function (Ueguchi et al., 1997), ultimately providing a less strenuous genetic landscape compared to full H-NS deletion. In all, our pipeline successfully identified a putative repressor for RseX, which we were able to validate by northern blotting. Notably, RseX was natively undetected for almost two decades since its discovery, highlighting the strength of this integrative approach, even for lowly-expressed sRNAs.

### Integrative Approach Uncovers Hidden Post-Transcriptional Regulation by sRNA RseX

**Figure 6:**
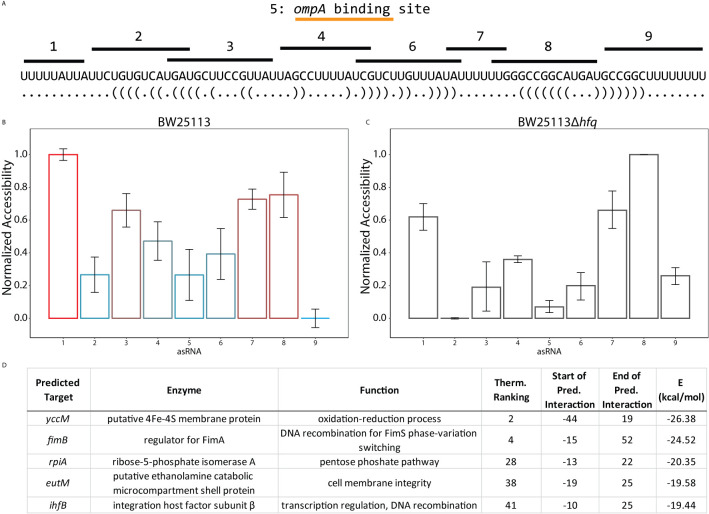
Target predictions informed by accessibility profiles suggest global post-transcriptional activity of RseX. **(A)** *In vivo* accessibility profile of RseX previously determined using a high throughput regional RNA accessibility quantification assay termed INTERFACE (Mihailovic et al., 2018). Targeted regions are indicated above the accepted RseX sequence. The region targeted by asRNA 5 corresponds to the predicted *ompA* binding site (Guillier et al., 2006). **(B)** RseX accessibility in WT *E*. *coli* BW25113 as collected in (Mihailovic et al., 2018). Results are normalized from 0 to 1 to allow for comparison across conditions (*i.e.*, varying abundance). Colors correspond to traditional visual representation of *in vivo* accessibility data (red = highly accessible, blue = lowly accessible). Error bars represent standard error of the mean. **(C)** RseX normalized accessibility in a *kanR-*cured isogenic Δ*hfq* strain (Baba et al., 2006). Likely-functional region 1 decreases accessibility (p-value < 0.05 2-tailed Student’s t-test) in the absence of match-maker Hfq, unlike likely-functional region 8. **(D)** Top-5 filtered target predictions of RseX at functional region 1. Two predicted targets, *ihfB* and *fimB*, were identified as most interesting given the newly confirmed global silencer (H-NS) of RseX, as both mRNAs encode for accepted transcriptional regulators. Start and end coordinates of putative RseX binding are listed for each mRNA with respect to translational start.

Because of the ability of RseX expression to enable survival under functional σ^E^ deficiency, we hypothesized that its entire target repertoire remains to be discovered. The notion of an expanded RseX sRNA targetome is further supported by the observation that some characterized sRNAs within the *E. coli* σ^E^ regulon (*e.g.*, RybB) have over 15 confirmed direct targets (Gogol et al., 2011). To date, RseX has been shown to post-transcriptionally regulate *ompA/C* in an Hfq-dependent manner (Douchin et al., 2006), likely via thermodynamically-predicted base-pairing within region 5, annotated in **Figure 6A** (Guillier et al., 2006). It is important to note that the low native expression of RseX (in cells producing H-NS) puts this sRNA at a disadvantage for competition with match-making proteins, explaining its limited representation in RIL-seq and CLASH interactome studies (see Methods for analysis details) (**Supplementary Data 4**) (Melamed et al., 2016; Waters et al., 2017; Iosub et al., 2020; Melamed et al., 2020). Thus, to expand our knowledge of the target regulation network of RseX, we derived RseX-specific insights from the ID-sRnA post-transcriptional activity node (**Figure 1B**). From this analysis, we selected RseX region 1 as a putative regulatory region based on its high accessibility that contrasts that of neighboring region 2 (**Figure 6B**). Lending further confidence to the selection of this region is the significant reduction in accessibility in an *hfq*-null strain (p-value < 0.05, 2-tailed t-test) (**Figure 6C**). Importantly, the ability of sRNAs to utilize multiple distinct portions of themselves for unique target binding activity is not unprecedented; this has been observed in multiple sRNAs including GcvB and FnrS (Durand and Storz, 2010; Lalaouna et al., 2019).

**Figure 7:**
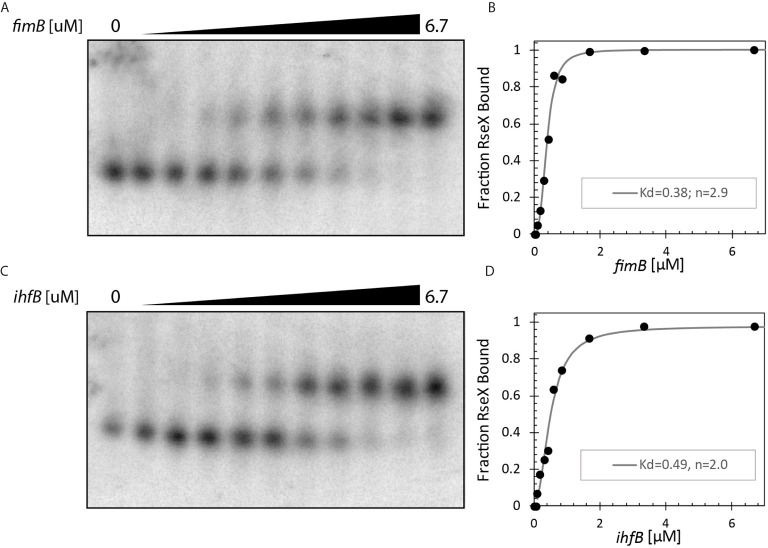
*In vitro* binding assays confirm direct interaction between RseX and **(A, B)**. *fimB* (-46 to +83 with respect to translational start) and **(C, D)**. *ihfB* (-53 to +70 with respect to translational start). 1.3 pmol RseX was included in each 12 μL binding reaction. Dissociation constants (K_d_) as shown in B/D were calculated using the modified Hill equations (Ryder et al., 2008). Notably, both targets have lower K_d_ values than previously reported target *ompA* (**Supplementary Figure 5**), suggesting stronger interactions for *fimB* and *ihfB*.

**Figure 8:**
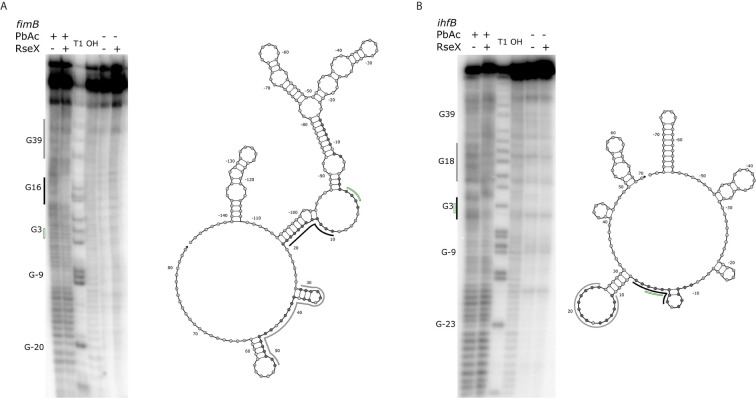
Results of PbAc probing of representative 5’-labeled *fimB* **(A)** and *ihfB* **(B)** transcripts confirm RseX protection in IntaRNA-predicted regions. Position of various G residues (as concluded from guanine “T1” and alkaline “OH” ladders) are labeled to the left of the probing images, numbered with respect to the start codon (green, no fill). Control reactions without PbAc indicate initial levels of cleavage. By comparing the mRNA levels of cleavage with and without RseX, regions exhibiting strong (black) and weak (grey) protection were identified as interaction sites and are outlined to the left of the probing images. Nucleotides thermodynamically predicted to interact with RseX (shaded), start codon (green, no fill), as well as corresponding regions of strong and weak RseX protection (black, grey traces) are overlaid on corresponding Nupack-predicted secondary structures (Zadeh et al., 2011) of the mRNA 5’ UTRs through the predicted RseX interaction sites.

**Figure 9:**
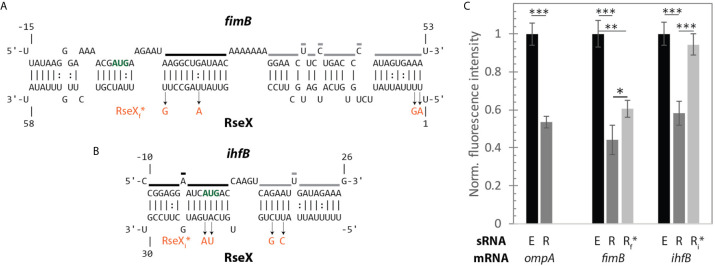
A fluorescent reporter system confirms direct translational regulation of mRNAs *fimB* and *ihfB* by RseX *in vivo*. **(A)** IntaRNA-predicted interaction between RseX and *fimB*. Start codon is outlined in bold green font; regions that are strongly (black) and weakly (grey) protected from cleavage in the presence of RseX, as determined by PbAc probing, are traced. RseX sequence mutations used in reporter assays, designed to limit changes in predicted structure, are listed in orange. **(B)** IntaRNA-predicted interaction between RseX and *ihfB*. Start codon, *in vitro* RseX-protected regions, and point mutations are outlined as in panel **(A)**. **(C)** *gfp* assays elucidate repressive *in vivo* effects of RseX on previously-confirmed (*ompA*) and novel (*fimB, ihfB*) targets at OD_600_ 1. Δ*rseX* strains harboring pNM12 (black), pBAD-RseX (dark grey) or pBAD-RseX mutant (light grey, RseX_f_* or RseX_i_* for *fimB* and *ihfB*, respectively) were induced by addition of 0.05% arabinose at OD_600_ ~ 0.15; respective pLacO-*ompA/fimB/ihfB-gfp* constructs were simultaneously induced with 1 mM IPTG. Illustrated means represent median fluorescence as normalized to respective pNM12 controls; samples for each median were collected in at least triplicate. Error bars represent propagated standard deviation of the mean and asterisks indicate significant differences as evaluated by unpaired Student’s t-test (p-value < 0.001, < 0.01, < 0.05 are represented as ***, **, and *, respectively). Positive control *ompA* as well as novel targets *fimB* and *ihfB* are repressed upon RseX expression, as compared to an empty control (p-value < 0.001). Repression of *fimB* by RseX is alleviated partially by 4 point mutations in RseX (RseX_f_*) outlined in **(A)** (p-value < 0.05). Repression of *ihfB* by RseX is fully abolished by 4 point mutations in RseX (RseX_i_*) (p-value < 0.001).

Upon constraining RseX-specific target predictions to its accessibility-inferred putative binding site, we considered the top-5 potential targets (**Figure 6D**), *yccM, fimB, rpiA, eutM, ihfB*. We hypothesized that suppression of RseP is enabled by global RseX activity, in which RseX regulates the expression of important regulators beyond known outer membrane proteins. In accord with this hypothesis, we select potential targets with known transcriptional regulation activity, *i.e.*, *fimB*, and *ihfB.* Importantly, we confirmed that RseX interacts with both *fimB* (**Figures 7A, B**) and *ihfB* (**Figures 7C, D** *)* (K_d_ 0.38 and 0.49 nM for 0.11 nM RseX) transcripts via *in vitro* electrophoretic mobility shift assays (EMSAs). Interestingly, our results show that the affinities of RseX for *fimB* and *ihfB* are higher than that of previously-confirmed target *ompA* (Kd 1.1 nM, **Supplementary Figure 5**). We further validate RseX-*fimB* and RseX-*ihfB* interactions, as predicted by IntaRNA, via lead acetate (PbAc) probing, revealing regions within 5’-labeled representative mRNA sequences that are protected from cleavage in the presence of RseX (**Figures 8A, B**, respectively). For both *fimB* and *ihfB*, this probing confirms the most-protected binding sites as those proximal to the start codon (black trace, **Figure 8** and **Figures 9A, B**). Importantly, only slight protection is observed at the 3’-most sites predicted to interact with the ID-sRnA-selected likely functional region of RseX (grey trace); this further supports a role for this highly accessible region (**Figure 6B**) in serving as a toehold for a stronger interaction.

To test for the regulatory significance of these interactions *in vivo*, we next performed *gfp*-based reporter assays in a K-12 Δ*rseX* strain using inducible mRNA-*gfp* (pBTRK-derivative plasmid) and sRNA (pNM12-based plasmid) expression (**Figure 9**). Importantly, the pBTRK is a low copy plasmid (1-3 copies at OD 0.4 in glucose-supplemented LB) (Youngquist et al., 2013) and, in this way, enables near-native copy numbers of the corresponding synthetic mRNA-GFP constructs, namely *ompA* (well-established target), *fimB*, and *ihfB*. As expected in the case of the *ompA-gfp* control, *ompA* levels were repressed in the RseX-overexpressed strain (via plasmid pBAD-RseX, whose strong induction was confirmed via northern blotting, **Supplementary Figure 6**), relative to the empty pNM12 plasmid (E) (no RseX) control (p-value < 0.001). This is consistent with known mechanisms of RseX-*ompA* repression, as supported by previous northern blotting upon RseX overexpression (Douchin et al., 2006). Significant repression was also observed in the case of *fimB*-*gfp and ihB*-*gfp* (p-value < 0.001) upon expression of WT RseX (R) relative to empty plasmid control (E) at OD_600_ 1 (**Figure 9C**); this was largely expected based on predicted RseX-mediated occlusion of the RBS and start codon, respectively (**Figures 9A, B**). It is worth noting that the magnitude of repression of both targets is comparable to that of known target *ompA*.

For each proposed novel target, we also designed and tested target-specific RseX mutations (RseX_f_* for *fimB* and RseX_i_ * for *ihfB*) in an attempt to limit interaction with confirmed RseX-protected regions (**Figure 8**). Four unique point mutations corresponding to two most-stable predicted consecutive interacting regions were selected for each RseX mutant, RseX_f_* and RseX_i_* (**Figures 9A, B**); final sequences were chosen based on minimization of changes within the predicted secondary structure. Importantly, RseX mutants that abolish interactions with *ompA* were not constructed given that specific RseX-*ompA* binding sites have not been mapped. For both *fimB*-*gfp* and *ihfB-gfp*, we observed repression relief by corresponding point mutations to the RseX sequence (RseX_f_* and RseX_i_ *) (p-value < 0.05, 0.001, respectively) (**Figure 9C**). We hypothesize that diminished disruption to the RseX-*fimB* interaction relative to the RseX-*ihfB* interaction occurs due to the extremely stable RseX-*fimB* interactions, predicted to span over 50 nucleotides. Notably, in conditions of reduced RseX overexpression (arabinose 0.01% instead of 0.05%), full repression relief is achieved (**Supplementary Figure 7**).

To provide additional validation of the RseX-*fimB* and RseX-*ihfB* interactions *in vivo*, we attempted to construct compensatory *fimB* and *ihfB* mutations to re-establish interaction with RseX_f_* and RseX_i_ *, respectively. However, given that predicted binding sites within *fimB* and *ihfB* involve coding sequence and occur at regions predicted to have high secondary structure, compensatory mutations were limited or entirely unfeasible, respectively. Indeed, in the case of *ihfB*, there were no viable compensatory mutations that met specified structure and codon frequency maintenance constraints. For *fimB*, one point mutation (of four total desired) met established mutation criteria and was predicted to partially reestablish regulation (**Supplementary Figure 7**). This “minimal” *fimB* mutant partially re-compensated repression by the corresponding RseX mutant, RseX_f_* (p-value < 0.05), although no significant differences on *fimB-gfp* mutant were detected between WT and RseX_f_* expression (**Supplementary Figure 7**). Altogether, these results confirm that the target repertoire of RseX is larger than previously appreciated and can be uncovered using the post-transcriptional node of the ID-sRnA approach.

## Discussion

Here we have developed a new approach, ID-sRnA, for the simultaneous analysis of multiple high throughput datasets to uncover putative regulators and targets of bacterial sRNAs. By incorporating multi-modal data collected under multiple environmental and genetic conditions, ID-sRnA can be used to capture the stress-responsive nature of sRNAs. We benchmark this fully-computational approach to showcase its ability to capture the sRNA contributions to larger transcriptional networks for a set of well-known sRNAs.

We additionally use ID-sRnA to identify H-NS as a negative regulator of RseX expression, a sRNA whose characterization within greater stress-response networks has been impeded for almost two decades due to its lack of known native expression conditions. Besides repressing the expression of hundreds of coding transcripts, including pathogenicity islands in *Salmonella*, H-NS has also been implicated in rapid post-transcriptional regulatory networks. For example, sRNAs MicF, GadF, and SsrS (6S) all have documented H-NS-dependent expression (Hör et al., 2020); furthermore, DsrA, an acid- and temperature-responsive sRNA (Lease et al., 2004), is known to rapidly downregulate *hns* in *E. coli* (Lalaouna et al., 2015). More recently, DsrA has been identified as a critical regulator for epithelial cell invasion in *Salmonella*, possibly owed to downstream de-repressive effects on virulence genes through its regulation of *hns* (Ryan et al., 2016). This suggests RseX may serve as a part of a larger stress response network in response to harsh host conditions. Aside from the unwinding of condensed DNA with decreased abundance of H-NS, it is likely that an activator is required to enable RseX expression. It is possible that the identified MarA/SoxS/Rob motifs near and within the 5’ RseX sequence acts as an activation site; potential RseX regulation by the antibiotic-resisting MarA/SoxS/Rob regulon agrees with previous studies that observe RseX overexpression improving cefalotin resistance (Kim et al., 2015). Interestingly, the post-transcriptional ID-sRnA pipeline further suggests RseX regulation that may be relevant in host-relevant stress response. Specifically, the discovery of type I fimbrial switch (FimS) regulator, *fimB*, as an RseX target supports previously identified phenotypic effects of RseX overexpression on biofilm formation and cell motility (Bak et al., 2015), Notably, a different fimbrial mRNA, *fimZ*, has previously been identified as an RseX binding partner in previous microarray analyses (Douchin et al., 2006), suggesting an broader RseX role in fimbrial-modulated epithelial attachment and perhaps colonization (Schwan et al., 2002). It will be interesting to further investigate a colonization role of RseX between different Enterobacteriaceae, including *Shigella* and *Citrobacter* sp (Nawrocki et al., 2015).

**Figure 10:**
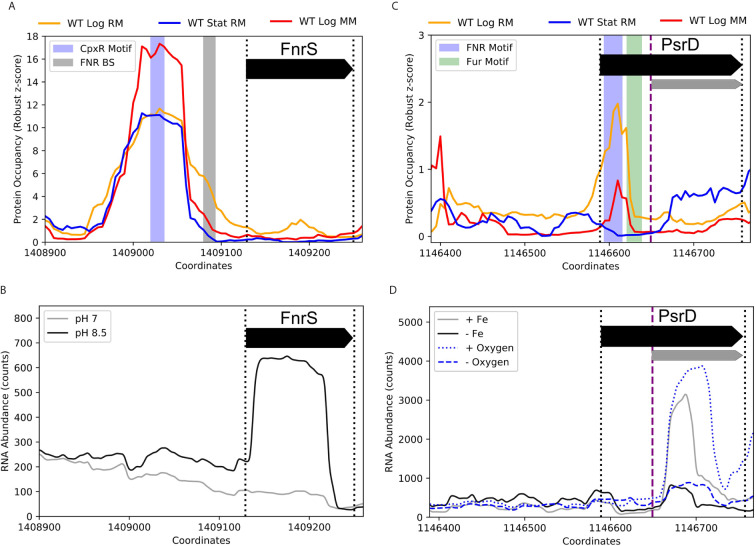
Promising novel DBPs suggest FnrS and PsrD play wider roles in stress response networks. **(A)** A CpxR motif was identified within a strong PO peak near the promoter of FnrS. CpxR is activated in response to inner membrane disruption, such as conditions of alkaline pH and high osmolarity. **(B)** RNA expression data (Gao et al., 2018) illustrate FnrS induction in alkaline conditions, supporting CpxR as a potential regulator of FnrS. **(C)** PsrD, an sRNA with no documented function, contains both FNR and Fur motifs within the annotated coding region near an embedded secondary promoter (FNR: +5 to +26 of accepted PsrD TSS, -55 to -34 of alternate TSS; Fur: +32 to +49 accepted TSS, -28 to –11 of alternate TSS). **(D)** RNA-seq counts (Seo et al., 2014) [GSE72113] highlight PsrD transcription induction under iron-rich and aerobic conditions (as compared to iron-poor and anaerobic conditions, respectively) and additionally showcase the activity of the secondary promoter (purple dashed line) that is ideally positioned to be regulated by the proposed FNR and Fur binding sites. Full-length transcripts have previously been detected at late stationary growth phases (Argaman et al., 2001).

Beyond RseX, multiple sRNAs had exciting potential transcriptional regulation uncovered by the ID-sRnA pipeline, ripe for experimental follow-up. Indeed, 163 DBPs corresponding to 62 sRNAs were designated as high confidence (bolded in **Supplementary Data 2**). Importantly, many of these present potentially yet-undiscovered regulation for sRNAs with known regulators. For example, FnrS has a striking differential PO peak located near the promoter region, shown in **Figure 10A**, that does not align with the known binding site of the anaerobic-responsive FNR. Rather, the center of the peak harbors a motif for a key player in the Cpx two-component envelope stress response system, CpxR. CpxR is phosphorylated under a variety of conditions in response to inner membrane disruption, including alkaline pH and high osmolarity (Hunke et al., 2012). In support of the proposed regulation by CpxR, FnrS was found to be significantly induced in high pH conditions (log2FC of 3.95, p-adj of 3.88e-10) (**Figure 10B**) (Gao et al., 2018). Interestingly, the third predicted target corresponding to the identified function region [84,95] encodes for inner membrane protein *yohJ* (**Supplementary Data 4**), aligning with the accepted CpxR role in mitigating envelope stress via regulation of inner membrane composition.

PsrD (also known as SraB), a confirmed sRNA with no documented function to date, is another notable instance in which the ID-sRnA pipeline offers compelling putative regulators and targets that may be worth experimental validation. The transcriptional node of the pipeline suggests both Fur and FNR as high-confidence regulators of a short, alternative transcript likely initiated by an internal promoter 60 nt from the accepted 5’ TSS (**Figure 10C**). Indeed, a ~105 nt alternate PsrD transcript has previously been observed in both log and stationary phase growth; additionally, transcription termination read-through has been proposed in log growth (Argaman et al., 2001). Interestingly, protein occupancy peaks corresponding to the Fur and FNR motif locations are observed downstream of the accepted TSS only in log growth (RM SNR > 2.5, MM SNR > 0.5), overlapping the promoter region of the alternate TSS (-28 to –11 and -55 to -34, respectively). Supporting the influence of the Fur and FNR regulators on PsrD expression, the alternate-PsrD product is significantly downregulated in iron-depleted conditions as well as under oxygen-limited biofilm conditions (**Figure 10D**). It is also worth noting that network links between divalent cation regulation and oxygen levels have been previously established (Beauchene et al., 2017). Furthermore, many top-5 filtered targets (*wecA*, *hypE*, *cusS* and *narU*) corresponding to two likely-functional PsrD regions (**Supplementary Data 4**) have established links to cation binding and/or regulation (*e.g.*, copper/silver export) as well as to anaerobic regulation (*e.g.*, nitrate/nitrite transport). We anticipate that using insights from the sRnA-ID pipeline to characterize alternate transcription and associated regulators of an unknown sRNA is one of many utilities of the large set of supplementary data.

When considering all proposed sRNA regulators, identification of putative DBP regulation within the accepted sRNA sequence (as opposed to upstream of the annotated TSS), was not isolated to PsrD. We detected putative internal motifs distal from promoters for several expected DBP-sRNA pairs (CRP-McaS, CpxR-CyaR, RpoS-GadY) within our high-confidence list. Perhaps their characterized DBP-sRNA regulation is augmented by these additional, non-traditional sites. Indeed, this has been observed of the well-studied LacI-*lac* operon regulation in which a LacI site located hundreds of nucleotides downstream from the promoter contributes to activation site blocking via DNA looping (Oehler et al., 1990). Furthermore, transcription termination efficiency at Rho-dependent terminators can be increased via H-NS-enabled colocalized DNA supercoiling (Kotlajich et al., 2015). Although DBP-enabled termination regulation at Rho-independent terminators has not yet been characterized in bacteria, transcriptional antitermination of sRNAs has been established as a sRNA-regulating mechanism; for instance, transcriptional read-through of DsrA and SgrS is reduced at low temperatures and under glucose-phosphate stress, respectively, enabling the accumulation of functional sRNA (Chen et al., 2019). However, associated regulators are not known, nor whether the mechanism of termination efficiency is due to interactions on the DNA or RNA level. Many high-confidence regulator-sRNA pairs picked up by this study potentially act *via* sRNA termination regulation, as inferred based on DBP motif location (*e.g.*, Fur-GlmY, Fur-IpeX, CRP-RyeG, Ada-FnrS, **Supplementary Data 2**). These pairs may merit further molecular characterization to understand potential contributions to DNA looping, RNAP pausing, disruption of RNAP-sigma factor interactions (Chen et al., 2019) or antiterminator complex formation (Santangelo and Artsimovitch, 2011) to affect sRNA termination.

We broadly anticipate that the proposed ID-sRnA approach will be useful for sRNA-reliant network characterization in all bacteria; however, we recognize its limited utility in organisms for which there is not an abundance of high throughput PO, RNA expression, or RNA accessibility and interactome data. Even within selected *E. coli* datasets, it is likely that many sRNAs under examination have multiple, environment-dependent regulation and activity that may not be captured in the considered IPOD-HR or INTERFACE conditions. Similarly, desired RNA-seq data was sometimes inaccessible due to lack of relevant deletion strains or reliance on sample preparation methods causing inadequate resolution of sRNAs. In light of these perceived limitations, it is important to note that the approach is amenable to modifications or exclusions at various steps. For example, in the absence of IPOD-HR protein occupancy data, motifs could be predicted corresponding to promoter regions only. We expect the utility of this approach to expand to more organisms with higher accuracy as more omics data elucidating conditional DBP-DNA interactions, RNA expression, and regional accessibility become available. Such investigation under pathologically-relevant environmental conditions will enable us to deduce complex rapid-regulation schemes that support infection.

## Data Availability Statement

Publicly available datasets were analyzed in this study. This data can be found here: Gene Expression Omnibus (GEO): GSE65642, GSE48324, GSE72113, GSE141694, GSE64848, GSE73672, GSE54900, GSE123554, GSE41190, GSE66481, GSE74809, GSE135516, GSE40313, GSE128611, GSE60522, GSE88980, GSE65711, GSE111094, GSE114917, GSE117939, GSE142291.

## Author Contributions

MM, AE, AL, BL, and JG designed and implemented the proposed approach. MM, AE, LC, and PF designed the approach and follow-up experiments (AC, EM). MM, AE, AC, BL, ML, and CE performed wet-lab experiments. MM and AE wrote the manuscript and LC, AL, PF, and EM provided manuscript edits. LC provided the direction and guidance for the project. All authors contributed to the article and approved the submitted version.

## Funding

This research was supported by the Welch Foundation (F-1756 to L.M.C.), National Science Foundation (MCB-1932780 to LC and DGE-1610403 to MM, AL, and AC), NIH (R35 GM128637 to PF), and Canadian Institutes of Health Research (CIHR to ÉM). We would further like to acknowledge the Cyberinfrastructure Research 4 Social Change Research Experiences for Undergraduates, made possible with support from National Science Foundation Award (#1852538 to JG).

## Conflict of Interest

The authors declare that the research was conducted in the absence of any commercial or financial relationships that could be construed as a potential conflict of interest.

## References

[B1] Ali Azam T. Iwata A. Nishimura A. Ueda S. Ishihama A. (1999). Growth Phase-Dependent Variation in Protein Composition of the Escherichia Coli Nucleoid. J. Bacteriol. 181 (20), 6361–6370. 10.1128/JB.181.20.6361-6370.1999 10515926 PMC103771

[B2] Anders S. Pyl P. T. Huber W. (2015). Htseq—a Python Framework to Work With High-Throughput Sequencing Data. Bioinformatics 31 (2), 166–169. 10.1093/bioinformatics/btu638 25260700 PMC4287950

[B3] Argaman L. Hershberg R. Vogel J. Bejerano G. Wagner E. G. H. Margalit H.. (2001). Novel Small RNA-Encoding Genes in the Intergenic Regions of Escherichia Coli. Curr. Biol. 11 (12), 941–950. 10.1016/S0960-9822(01)00270-6 11448770

[B4] Azam M. S. Vanderpool C. K. (2020). Translation Inhibition From a Distance: The Small RNA Sgrs Silences a Ribosomal Protein S1-Dependent Enhancer. Mol. Microbiol. 114 (3), 391–408. 10.1111/mmi.14514 32291821 PMC7502529

[B5] Baba T. Ara T. Hasegawa M. Takai Y. Okumura Y. Baba M.. (2006). Construction of Escherichia Coli K-12 in-Frame, Single-Gene Knockout Mutants: The Keio Collection. Mol. Syst. Biol. 2, 2006.0008–2006.0008. 10.1038/msb4100050 PMC1681482 16738554

[B6] Bak G. Lee J. Suk S. Kim D. Young Lee J. Kim K.-S.. (2015). Identification of Novel Srnas Involved in Biofilm Formation, Motility, and Fimbriae Formation in Escherichia Coli. Sci. Rep. 5, 15287. 10.1038/srep15287 26469694 PMC4606813

[B7] Barquist L. Vogel J. (2015). Accelerating Discovery and Functional Analysis of Small Rnas With New Technologies. Annu. Rev. Genet. 49 (1), 367–394. 10.1146/annurev-genet-112414-054804 26473381

[B8] Beauchene N. A. Mettert E. L. Moore L. J. Keleş S. Willey E. R. Kiley P. J. (2017). O(2) Availability Impacts Iron Homeostasis in Escherichia Coli. Proc. Natl. Acad. Sci. U S A 114 (46), 12261–12266. 10.1073/pnas.1707189114 29087312 PMC5699043

[B9] Bhatt S. Egan M. Ramirez J. Xander C. Jenkins V. Muche S.. (2017). Hfq and Three Hfq-Dependent Small Regulatory Rnas-Mgrr, Ryhb and Mcas-Coregulate the Locus of Enterocyte Effacement in Enteropathogenic Escherichia Coli. Pathog. Dis. 75 (1), ftw113. 10.1093/femspd/ftw113 27956465 PMC5827581

[B10] Bossi L. Figueroa-Bossi N. Bouloc P. Boudvillain M. (2020). Regulatory Interplay Between Small Rnas and Transcription Termination Factor Rho. Biochim. Biophys. Acta (BBA) - Gene Regul. Mech. 1863 (7), 194546. 10.1016/j.bbagrm.2020.194546 32217107

[B11] Bowman E. K. Mihailovic M. K. Li B. Contreras L. M. (2020). Bioinformatic Application of Fluorescence-Based In Vivo RNA Regional Accessibility Data to Identify Novel Srna Targets. Methods Mol. Biol. 2113, 41–71. 10.1007/978-1-0716-0278-2_5 32006307

[B12] Carrier M.-C. Lalaouna D. Massé E. (2016). A Game of Tag: MAPS Catches Up on RNA Interactomes. RNA Biol. 13 (5), 473–476. 10.1080/15476286.2016.1156830 26967018 PMC4962807

[B13] Castillo-Keller M. Vuong P. Misra R. (2006). Novel Mechanism of Escherichia Coli Porin Regulation. J. Bacteriol. 188 (2), 576–586. 10.1128/JB.188.2.576-586.2006 16385048 PMC1347279

[B14] Chakravarty S. Massé E. (2019). RNA-Dependent Regulation of Virulence in Pathogenic Bacteria. Front. Cell. Infection Microbiol. 9, 337. 10.3389/fcimb.2019.00337 PMC6794450 31649894

[B15] Chen S. Lesnik E. A. Hall T. A. Sampath R. Griffey R. H. Ecker D. J.. (2002). A Bioinformatics Based Approach to Discover Small RNA Genes in the Escherichia Coli Genome. Biosystems 65 (2-3), 157–177. 10.1016/s0303-2647(02)00013-8 12069726

[B16] Chen Z. Lewis K. A. Shultzaberger R. K. Lyakhov I. G. Zheng M. Doan B.. (2007). Discovery of Fur Binding Site Clusters in Escherichia Coli by Information Theory Models. Nucleic Acids Res. 35 (20), 6762–6777. 10.1093/nar/gkm631 17921503 PMC2189734

[B17] Chen J. Morita T. Gottesman S. (2019). Regulation of Transcription Termination of Small Rnas and by Small Rnas: Molecular Mechanisms and Biological Functions. Front. Cell. Infection Microbiol. 9, 201. 10.3389/fcimb.2019.00201 PMC6582626 31249814

[B18] Clough E. Barrett T. (2016). The Gene Expression Omnibus Database. Methods Mol. Biol. (Clifton N.J.) 1418, 93–110. 10.1007/978-1-4939-3578-9_5 PMC4944384 27008011

[B19] Denham E. L. (2020). The Sponge Rnas of Bacteria – How to Find Them and Their Role in Regulating the Post-Transcriptional Network. Biochim. Biophys. Acta (BBA) - Gene Regul. Mech. 1863 (8), 194565. 10.1016/j.bbagrm.2020.194565 32475775

[B20] Desgranges E. Caldelari I. Marzi S. Lalaouna D. (2020). Navigation Through the Twists and Turns of RNA Sequencing Technologies: Application to Bacterial Regulatory Rnas. Biochim. Biophys. Acta (BBA) - Gene Regul. Mech. 1863 (3), 194506. 10.1016/j.bbagrm.2020.194506 32068131

[B21] Desnoyers G. Morissette A. Prevost K. Masse E. (2009). Small RNA-Induced Differential Degradation of the Polycistronic Mrna Iscrsua. EMBO J. 28, 1551–1561. 10.1038/emboj.2009.116 19407815 PMC2693151

[B22] Douchin V. Bohn C. Bouloc P. (2006). Down-Regulation of Porins by a Small RNA Bypasses the Essentiality of the Regulated Intramembrane Proteolysis Protease Rsep in Escherichia Coli. J. Biol. Chem. 281 (18), 12253–12259. 10.1074/jbc.M600819200 16513633

[B23] Durand S. Storz G. (2010). Reprogramming of Anaerobic Metabolism by the Fnrs Small RNA. Mol. Microbiol. 75 (5), 1215–1231. 10.1111/j.1365-2958.2010.07044.x 20070527 PMC2941437

[B24] Eckweiler D. Dudek C.-A. Hartlich J. Brötje D. Jahn D. (2018). PRODORIC2: The Bacterial Gene Regulation Database in 2018. Nucleic Acids Res. 46 (D1), D320–D326. 10.1093/nar/gkx1091 29136200 PMC5753277

[B25] Fang F. C. Rimsky S. (2008). New Insights Into Transcriptional Regulation by H-NS. Curr. Opin. Microbiol. 11 (2), 113–120. 10.1016/j.mib.2008.02.011 18387844 PMC2394665

[B26] Fozo E. M. Kawano M. Fontaine F. Kaya Y. Mendieta K. S. Jones K. L.. (2008). Repression of Small Toxic Protein Synthesis by the Sib and Ohsc Small Rnas. Mol. Microbiol. 70 (5), 1076–1093. 10.1111/j.1365-2958.2008.06394.x 18710431 PMC2597788

[B27] Freddolino P. L. Amemiya H. M. Goss T. J. Tavazoie S. (2021). Dynamic Landscape of Protein Occupancy Across the Escherichia coli Chromosome. PLoS Biol. 19 (6), e3001306. 10.1371/journal.pbio.3001306 34170902 PMC8282354

[B28] Gama-Castro S. Salgado H. Peralta-Gil M. Santos-Zavaleta A. Muniz-Rascado L. Solano-Lira H.. (2011). Regulondb Version 7.0: Transcriptional Regulation of Escherichia Coli K-12 Integrated Within Genetic Sensory Response Units (Gensor Units). Nucleic Acids Res. 39, D98–D105. 10.1093/nar/gkq1110 21051347 PMC3013702

[B29] Gama-Castro S. Salgado H. Santos-Zavaleta A. Ledezma-Tejeida D. Muñiz-Rascado L. García-Sotelo J. S.. (2016). Regulondb Version 9.0: High-Level Integration of Gene Regulation, Coexpression, Motif Clustering and Beyond. Nucleic Acids Res. 44 (D1), D133–D143. 10.1093/nar/gkv1156 26527724 PMC4702833

[B30] Gao Y. Yurkovich J. T. Seo S. W. Kabimoldayev I. Dräger A. Chen K.. (2018). Systematic Discovery of Uncharacterized Transcription Factors in Escherichia Coli K-12 Mg1655. Nucleic Acids Res. 46 (20), 10682–10696. 10.1093/nar/gky752 30137486 PMC6237786

[B31] Gimpel M. Brantl S. (2017). Dual-Function Small Regulatory Rnas in Bacteria. Mol. Microbiol. 103 (3), 387–397. 10.1111/mmi.13558 27750368

[B32] Gogol E. B. Rhodius V. A. Papenfort K. Vogel J. Gross C. A. (2011). Small Rnas Endow a Transcriptional Activator With Essential Repressor Functions for Single-Tier Control of a Global Stress Regulon. Proc. Natl. Acad. Sci. U.S.A. 108 (31), 12875–12880. 10.1073/pnas.1109379108 21768388 PMC3150882

[B33] Gottesman S. (2019). Trouble is Coming: Signaling Pathways That Regulate General Stress Responses in Bacteria. J. Biol. Chem. 294 (31), 11685–11700. 10.1074/jbc.REV119.005593 31197038 PMC6682744

[B34] Grant C. E. Bailey T. L. Noble W. S. (2011). FIMO: Scanning for Occurrences of a Given Motif. Bioinformatics 27 (7), 1017–1018. 10.1093/bioinformatics/btr064 21330290 PMC3065696

[B35] Guillier M. Gottesman S. Storz G. (2006). Modulating the Outer Membrane With Small Rnas. Genes Dev. 20, 2338–2348. 10.1101/gad.1457506 16951250

[B36] Haning K. Engels S. M. Williams P. Arnold M. Contreras L. M. (2020). Applying a New REFINE Approach in Zymomonas Mobilis Identifies Novel sRNAs That Confer Improved Stress Tolerance Phenotypes. Front. Microbiol. 10, 2987. 10.3389/fmicb.2019.02987 31998271 PMC6970203

[B37] Hobbs E. C. Astarita J. L. Storz G. (2010). Small RNAs and Small Proteins Involved in Resistance to Cell Envelope Stress and Acid Shock in Escherichia Coli: Analysis of a Bar-Coded Mutant Collection. J. Bacteriol. 192 (1), 59–67. 10.1128/jb.00873-09 19734312 PMC2798238

[B38] Holmqvist E. Berggren S. Rizvanovic A. (2020). RNA-Binding Activity and Regulatory Functions of the Emerging sRNA-Binding Protein Proq. Biochim. Biophys. Acta (BBA) - Gene Regul. Mech. 1863 (9), 194596. 10.1016/j.bbagrm.2020.194596 32565402

[B39] Holmqvist E. Wagner E. G. H. (2017). Impact of Bacterial Srnas in Stress Responses. Biochem. Soc. Trans. 45 (6), 1203–1212. 10.1042/bst20160363 29101308 PMC5730939

[B40] Hör J. Gorski S. A. Vogel J. (2018). Bacterial RNA Biology on a Genome Scale. Mol. Cell 70 (5), 785–799. 10.1016/j.molcel.2017.12.023 29358079

[B41] Hör J. Matera G. Vogel J. Gottesman S. Storz G. (2020). Trans-Acting Small Rnas and Their Effects on Gene Expression in Escherichia Coli and Salmonella Enterica. EcoSal Plus 9 (1), 1–24. 10.1128/ecosalplus.ESP-0030-2019 PMC7112153 32213244

[B42] Hör J. Vogel J. (2017). Global Snapshots of Bacterial RNA Networks. EMBO J. 36 (3), 245–247. 10.15252/embj.201696072 28031253 PMC5286387

[B43] Hunke S. Keller R. Müller V. S. (2012). Signal Integration by the Cpx-Envelope Stress System. FEMS Microbiol. Lett. 326 (1), 12–22. 10.1111/j.1574-6968.2011.02436.x 22092888

[B44] Iosub I. A. van Nues R. W. McKellar S. W. Nieken K. J. Marchioretto M. Sy B.. (2020). Hfq CLASH Uncovers sRNA-Target Interaction Networks Linked to Nutrient Availability Adaptation. eLife 9, e54655. 10.7554/eLife.54655 32356726 PMC7213987

[B45] Jørgensen M. G. Pettersen J. S. Kallipolitis B. H. (2020). Srna-Mediated Control in Bacteria: An Increasing Diversity of Regulatory Mechanisms. Biochim. Biophys. Acta (BBA) - Gene Regul. Mech. 1863 (5), 194504. 10.1016/j.bbagrm.2020.194504 32061884

[B46] Jørgensen M. G. Thomason M. K. Havelund J. Valentin-Hansen P. Storz G. (2013). Dual Function of the Mcas Small RNA in Controlling Biofilm Formation. Genes Dev. 27 (10), 1132–1145. 10.1101/gad.214734.113 23666921 PMC3672647

[B47] Jose B. R. Gardner P. P. Barquist L. (2019). Transcriptional Noise and Exaptation as Sources for Bacterial Srnas. Biochem. Soc. Trans. 47 (2), 527–539. 10.1042/bst20180171 30837318

[B48] Keseler I. M. Collado-Vides J. Santos-Zavaleta A. Peralta-Gil M. Gama-Castro S. Muniz-Rascado L.. (2011). Ecocyc: A Comprehensive Database of Escherichia Coli Biology. Nucleic Acids Res. 39, D583–D590. 10.1093/nar/gkq1143 21097882 PMC3013716

[B49] Kim T. Bak G. Lee J. Kim K.-S. (2015). Systematic Analysis of the Role of Bacterial Hfq-Interacting Srnas in the Response to Antibiotics. J. Antimicrobial Chemotherapy 70 (6), 1659–1668. 10.1093/jac/dkv042 25724987

[B50] Kotlajich M. V. Hron D. R. Boudreau B. A. Sun Z. Lyubchenko Y. L. Landick R. (2015). Bridged Filaments of Histone-Like Nucleoid Structuring Protein Pause RNA Polymerase and Aid Termination in Bacteria. eLife 4, e04970. 10.7554/eLife.04970 PMC4337669 25594903

[B51] Lalaouna D. Eyraud A. Devinck A. Prévost K. Massé E. (2019). GcvB Small RNA Uses Two Distinct Seed Regions to Regulate an Extensive Targetome. Mol. Microbiol. 111 (2), 473–486. 10.1111/mmi.14168 30447071

[B52] Lalaouna D. Morissette A. Carrier M.-C. Massé E. (2015). DsrA Regulatory RNA Represses Both Hns and RbsD mRNAs Through Distinct Mechanisms in Escherichia Coli. Mol. Microbiol. 98 (2), 357–369. 10.1111/mmi.13129 26175201

[B53] Lease R. A. Smith D. McDonough K. Belfort M. (2004). The Small Noncoding Dsra RNA is an Acid Resistance Regulator in Escherichia Coli. J. Bacteriol 186 (18), 6179–6185. 10.1128/jb.186.18.6179-6185.2004 15342588 PMC515158

[B54] Leonard S. Meyer S. Lacour S. Nasser W. Hommais F. Reverchon S. (2019). APERO: A Genome-Wide Approach for Identifying Bacterial Small Rnas From RNA-Seq Data. Nucleic Acids Res. 47 (15), e88–e88. 10.1093/nar/gkz485 31147705 PMC6735904

[B55] Li H. Durbin R. (2010). Fast and Accurate Long-Read Alignment With Burrows-Wheeler Transform. Bioinformatics 26 (5), 589–595. 10.1093/bioinformatics/btp698 20080505 PMC2828108

[B56] Love M. I. Huber W. Anders S. (2014). Moderated Estimation of Fold Change and Dispersion for RNA-Seq Data With Deseq2. Genome Biol. 15 (12), 550. 10.1186/s13059-014-0550-8 25516281 PMC4302049

[B57] Majdalani N. Chen S. Murrow J. St John K. Gottesman S. (2001). Regulation of RpoS by a Novel Small RNA: The Characterization of Rpra. Mol. Microbiol. 39 (5), 1382–1394. 10.1111/j.1365-2958.2001.02329.x 11251852

[B58] Mann M. Wright P. R. Backofen R. (2017). Intarna 2.0: Enhanced and Customizable Prediction of RNA–RNA Interactions. Nucleic Acids Res. 45 (Web Server issue), W435–W439. 10.1093/nar/gkx279 28472523 PMC5570192

[B59] Martin M. (2011). CUTADAPT Removes Adapter Sequences From High-Throughput Sequencing Reads. EMBnet J 17 (1), 10–12. 10.14806/ej.17.1.200

[B60] McClune C. J. Alvarez-Buylla A. Voigt C. A. Laub M. T. (2019). Engineering Orthogonal Signalling Pathways Reveals the Sparse Occupancy of Sequence Space. Nature 574 (7780), 702–706. 10.1038/s41586-019-1639-8 31645757 PMC6858568

[B61] Mediati D. G. Wu S. Wu W. Tree J. J. (2020). Networks of Resistance: Small RNA Control of Antibiotic Resistance. Trends Genet. 10.1016/j.tig.2020.08.016 32951948

[B62] Melamed S. Adams P. P. Zhang A. Zhang H. Storz G. (2020). RNA-RNA Interactomes of Proq and Hfq Reveal Overlapping and Competing Roles. Mol. Cell 77 (2), 411–425.e417. 10.1016/j.molcel.2019.10.022 31761494 PMC6980735

[B63] Melamed S. Peer A. Faigenbaum-Romm R. Gatt Y. E. Reiss N. Bar A.. (2016). Global Mapping of Small RNA-Target Interactions in Bacteria. Mol. Cell 63 (5), 884–897. 10.1016/j.molcel.2016.07.026 27588604 PMC5145812

[B64] Mihailovic M. K. Vazquez-Anderson J. Li Y. Fry V. Vimalathas P. Herrera D.. (2018). High-Throughput *In Vivo* Mapping of RNA Accessible Interfaces to Identify Functional sRNA Binding Sites. Nat. Commun. 9 (1), 1–16. 10.1038/s41467-018-06207-z 30287822 PMC6172242

[B65] Modi S. R. Camacho D. M. Kohanski M. A. Walker G. C. Collins J. J. (2011). Functional Characterization of Bacterial Srnas Using a Network Biology Approach. Proc. Natl. Acad. Sci. U.S.A. 108 (37), 15522–15527. 10.1073/pnas.1104318108 21876160 PMC3174649

[B66] Moon K. Gottesman S. (2009). A Phoq/P-Regulated Small RNA Regulates Sensitivity of Escherichia Coli to Antimicrobial Peptides. Mol. Microbiol. 74 (6), 1314–1330. 10.1111/j.1365-2958.2009.06944.x 19889087 PMC2841474

[B67] Nawrocki E. P. Burge S. W. Bateman A. Daub J. Eberhardt R. Y. Eddy S. R.. (2015). Rfam 12.0: Updates to the RNA Families Database. Nucleic Acids Res. 43 (D1), D130–D137. 10.1093/nar/gku1063 25392425 PMC4383904

[B68] Oehler S. Eismann E. R. Krämer H. Müller-Hill B. (1990). The Three Operators of the Lac Operon Cooperate in Repression. EMBO J. 9 (4), 973–979. 10.1002/j.1460-2075.1990.tb08199.x 2182324 PMC551766

[B69] Pachkov M. Balwierz P. J. Arnold P. Ozonov E. van Nimwegen E. (2013). Swissregulon, a Database of Genome-Wide Annotations of Regulatory Sites: Recent Updates. Nucleic Acids Res. 41 (Database issue), D214–D220. 10.1093/nar/gks1145 23180783 PMC3531101

[B70] Raghavan R. Groisman E. A. Ochman H. (2011). Genome-Wide Detection of Novel Regulatory RNAs in E. Coli. Genome Res. 21, 1487–1497. 10.1101/gr.119370.110 21665928 PMC3166833

[B71] Robison K. McGuire A. M. Church G. M. (1998). A Comprehensive Library of DNA-Binding Site Matrices for 55 Proteins Applied to the Complete Escherichia Coli K-12 Genome. J. Mol. Biol. 284 (2), 241–254. 10.1006/jmbi.1998.2160 9813115

[B72] Ryan D. Ojha U. K. Jaiswal S. Padhi C. Suar M. (2016). The Small RNA Dsra Influences the Acid Tolerance Response and Virulence of Salmonella Enterica Serovar Typhimurium. Front. Microbiol. 7, 599. 10.3389/fmicb.2016.00599 27199929 PMC4844625

[B73] Ryder S. P. Recht M. I. Williamson J. R. (2008). Quantitative Analysis of Protein-RNA Interactions by Gel Mobility Shift. Methods Mol. Biol. 488, 99–115. 10.1007/978-1-60327-475-3_7 18982286 PMC2928675

[B74] Santangelo T. J. Artsimovitch I. (2011). Termination and Antitermination: RNA Polymerase Runs a Stop Sign. Nat. Rev. Microbiol. 9 (5), 319–329. 10.1038/nrmicro2560 21478900 PMC3125153

[B75] Santiago-Frangos Andrew W. S. A. (2018). Hfq Chaperone Brings Speed Dating to Bacterial sRNA. WIREs RNA 9, e1475. 10.1002/wrna.1475 29633565 PMC6002925

[B76] Schneider C. A. Rasband W. S. Eliceiri K. W. (2012). NIH Image to Imagej: 25 Years of Image Analysis. Nat. Methods 9 (7), 671–675. 10.1038/nmeth.2089 22930834 PMC5554542

[B77] Schwan W. R. Lee J. L. Lenard F. A. Matthews B. T. Beck M. T. (2002). Osmolarity and Ph Growth Conditions Regulate Fim Gene Transcription and Type 1 Pilus Expression in Uropathogenic Escherichia Coli. Infect. Immun. 70 (3), 1391–1402. 10.1128/iai.70.3.1391-1402.2002 11854225 PMC127777

[B78] Seo S. W. Kim D. Latif H. O’Brien E. J. Szubin R. Palsson B. O. (2014). Deciphering Fur Transcriptional Regulatory Network Highlights its Complex Role Beyond Iron Metabolism in Escherichia Coli. Nat. Commun. 5 (1), 4910. 10.1038/ncomms5910 25222563 PMC4167408

[B79] Sowa S. W. Gelderman G. Leistra A. N. Buvanendiran A. Lipp S. Pitaktong A.. (2017). Integrative Fourd Omics Approach Profiles the Target Network of the Carbon Storage Regulatory System. Nucleic Acids Res. 45 (4), 1673–1686. 10.1093/nar/gkx048 28126921 PMC5389547

[B80] Srinivasan R. Chandraprakash D. Krishnamurthi R. Singh P. Scolari V. F. Krishna S.. (2013). Genomic Analysis Reveals Epistatic Silencing of “Expensive” Genes in Escherichia Coli K-12. Mol. Biosyst. 9 (8), 2021–2033. 10.1039/c3mb70035f 23661089

[B81] Ueguchi C. Seto C. Suzuki T. Mizuno T. (1997). Clarification of the Dimerization Domain and its Functional Significance for the Escherichia Coli Nucleoid Protein H-NS 11Edited by I. B. Holland. J. Mol. Biol. 274 (2), 145–151. 10.1006/jmbi.1997.1381 9398522

[B82] Véscovi E. G. Ayala Y. M. Di Cera E. Groisman E. A. (1997). Characterization of the Bacterial Sensor Protein Phoq. Evidence for Distinct Binding Sites for Mg2+ and Ca2+. J. Biol. Chem. 272 (3), 1440–1443. 10.1074/jbc.272.3.1440 8999810

[B83] Villa J. K. Su Y. Contreras L. M. Hammond M. C. (2018). Synthetic Biology of Small Rnas and Riboswitches. Microbiol. Spectr. 6 (3), 1–18. 10.1128/microbiolspec.RWR-0007-2017 PMC6020158 29932045

[B84] Vogel J. Sharma C. M. (2005). How to Find Small non-Coding RNAs in Bacteria. Biol. Chem. 386 (12), 1219–1238. 10.1515/bc.2005.140 16336117

[B85] Waters S. A. McAteer S. P. Kudla G. Pang I. Deshpande N. P. Amos T. G.. (2017). Small RNA Interactome of Pathogenic E. Coli Revealed Through Crosslinking of Rnase E. EMBO J. 36 (3), 374–387. 10.15252/embj.201694639 27836995 PMC5286369

[B86] Yamada H. Yoshida T. Tanaka K. Sasakawa C. Mizuno T. (1991). Molecular Analysis of the Escherichia Coli Hns Gene Encoding a DNA-Binding Protein, Which Preferentially Recognizes Curved DNA Sequences. Mol. Gen. Genet. 230 (1-2), 332–336. 10.1007/bf00290685 1745240

[B87] Yin X. Wu Orr M. Wang H. Hobbs E. C. Shabalina S. A. Storz G. (2019). The Small Protein Mgts and Small RNA MgrR Modulate the Pita Phosphate Symporter to Boost Intracellular Magnesium Levels. Mol. Microbiol. 111 (1), 131–144. 10.1111/mmi.14143 30276893 PMC6351178

[B88] Youngquist J. T. Schumacher M. H. Rose J. P. Raines T. C. Politz M. C. Copeland M. F.. (2013). Production of Medium Chain Length Fatty Alcohols From Glucose in Escherichia Coli. Metab. Eng. 20, 177–186. 10.1016/j.ymben.2013.10.006 24141053 PMC3866921

[B89] Zadeh J. N. Steenberg C. D. Bois J. S. Wolfe B. R. Pierce M. B. Khan A. R.. (2011). NUPACK: Analysis and Design of Nucleic Acid Systems. J. Comput. Chem. 32 (1), 170–173. 10.1002/jcc.21596 20645303

[B90] Zere T. R. Vakulskas C. A. Leng Y. Pannuri A. Potts A. H. Dias R.. (2015). Genomic Targets and Features of Bara-Uvry (-SirA) Signal Transduction Systems. PloS One 10 (12), e0145035. 10.1371/journal.pone.0145035 26673755 PMC4682653

