# Supplementary material for: Uncovering Transcriptional Regulators and Targets of sRNAs Using an Integrative Data-Mining Approach: H-NS-Regulated RseX as a Case Study
Source: Front Cell Infect Microbiol. 2021 Jul 13;11:696533. doi: 10.3389/fcimb.2021.696533 (PMC8313858; doi:10.3389/fcimb.2021.696533)
Supplement: Supplementary file 5 [file DataSheet_5.pdf]

Supplementary Material

1.1 Supplementary Data Descriptions

Supplementary Data 1. Summary of *E. coli* K-12 MG1655 sRNAs under investigation in the proposed ID-sRnA approach. List of all 91 sRNAs used in the ID-sRnA approach in *E. coli*, with associated genomic coordinates and direction as well as other relevant annotation information. Also included are short summaries of ID-sRnA outputs, including information on functional regions used in the post-transcriptional pipeline and number of DBPs found for each sRNA, among other details.

Supplementary Data 2. Compiled results from ID-sRnA transcriptional regulation node. Coordinates of differential PO peaks, identities of peak-corresponding DBP motifs, and differential expression conditions found for each sRNA. Bolded DBPs were supported by differential expression analysis (DBP-condition relationships used are outlined in second sheet), enabling placement in the high confidence set. Green DBPs match previous literature support for regulation of the given sRNA.

Supplementary Data 3. RNA-Seq details for differential expression analysis. Relevant details for differentially expressed sRNAs per condition analyzed, along with the GEO accession numbers and citations for the data-mined RNA-Seq datasets.

Supplementary Data 4. Results from post-transcriptional activity node. Listed (up to) top-5 targets corresponding to each functional region identified from previously-published accessibility data, along with any (i) documented interactome method-based support for the sRNA-mRNA interaction, as well as (ii) alignment of targets with accepted sRNA function, either uncovered by the ID-sRnA approach, or previously documented regulatory/Gene Ontology information.

1.2 Supplementary Tables

Supplementary Table 1. Relevant DNA oligonucleotides and gBlocks for experimental methods performed in this study. Details on oligonucleotides and gBlocks (IDT) used to enable *in vitro* binding assays, *in vivo* reporter assays, and northern blotting. Refer to Methods section for further details.

Primer Description	Fwd Primer	Rev Primer	Final Construct Name
RseX hybridization probe	GCTAATAACGGAAGCATCATGACACAG	N/A	N/A
5S hybridization probe	CGGCGCTACGGCGTTTCACTTCTG	N/A	N/A
amplification of RseX from MG1655 for IVT	GACTTAATACGACTCACTATAGGGAGATTTTTATTATTCTGTGTCATGATGCTTCCGTT	AAAAAAAAGCCGGCATCATGCC	N/A
amplification of fimB from MG1655 for IVT	GACTTAATACGACTCACTATAGGGAGATAACCCAGCACAGCTAG	CCGGTATTTGCTGCTTT	N/A
amplification of ihfB from MG1655 for IVT	GACTTAATACGACTCACTATAGGGAGAATCAATGCAGCAACAGCAGCC	CTTCAACCGTCTTGGCGGGAAT	N/A
amplification of ompA from MG1655 for IVT	TAATACGACTCACTATAGGGTCGCCAG GGGTGCTCGGC	GCCAGTGCCACTGCAATCGCGATA	N/A
amplification of pBTRK backbone for pLacO insertion	ATGAGAGAAGATTTTCAGCCTG	CAGCTCATTTCAGAATATTTGCC	pBTRK-pLacO-Empty
gBlock for insertion of pLacO+MCS into pBTRK	gcaaatattctgaaatgagctGATAAATGTGAGCGGATAACATTGACATTGTGAGCGGATAACAAGATACTgtcgacgaattcgagctcggtacccggggatcctctagagtcgacctgcaggcatgcaagcttggctgttttggcggatgagagaagattttcagcct	N/A	
amplification of pBTRK backbone for cmR to catR swap	cggggcgtaaGGGCGGATCCCCCTCAAG	gtgccgatcaCTACCGGCGCGGCAGC	intermediate-for-pBTRK-pLacO-Empty-CmR

amplification of catR from pBAD18cm for insertion to pBTRK-pLacO-Empty	gcgccggtagTGATCGGCACGTAAGAGGTTC	ggatccgcccTTACGCCCCGCCCTGC	
amplification of intermediate-for-pBTRK-pLacO-Empty-CmR from upstream of rrnB1 terminator to 5' of cmR	cttggctgttTAGGGAACTGCCAGGCATC	GAACCTCTTACGTGCCGATC	pBTRK-pLacO-Empty-CmR
amplification of intermediate-for-pBTRK-pLacO-Empty-CmR from 5' of cmR to 3' of MCS	TGATCGGCACGTAAGAGG	cagttccctaAACAGCCAAGCTTGCATGC	
amplification of GFP from pHL1756 for pBTRK_lacO_cmR insertion	AAAGGAGAAGAACTTTTCAC	agttccctaaacagccTTATTTGTATAGTTCATCCATGC	see next 4 rows
amplification of ompA for 3-piece Gibson assembly (with GFP) into pBTRK_lacO_cmR	agcggataacaagatactgtcgacACATCGCCAGGGGTGCTC	tgaaaagttcttctcctttGAAACCAGCCAGTGCCAC	pBTRK_lacO_cmR_ompA
amplification of fimB for 3-piece Gibson assembly (with GFP) into pBTRK_lacO_cmR	agcggataacaagatactgtcgacCTCCATTTAAGTTCACTCAGAAG	tgaaaagttcttctcctttATTACGTGCTGCATGAGG	pBTRK_lacO_cmR_fimB
amplification of ihfB for 3-piece Gibson assembly (with GFP) into pBTRK_lacO_cmR	agcggataacaagatactgtcgacACTAAGGGCGGCTACGGC	tgaaaagttcttctcctttTGCATCTTCAACCGTCTTGGC	pBTRK_lacO_cmR_ihfB

gBlock for insertion of fimB_minimal_mut +GFP into pBTRK_lacO_cmR_ihfB	agcggataacaagatactgtcgacCTCCATTTAAGTTCACTCAGAAGAACTGGTCCACTTACGTTAGTTATTAAGCAAACGTTCGCTTTTATAAACATAATCAGGATAAAAATGTTGGATTATTGCTAACCCAGCACAGCTAGTGCGCGTCTGTAATTATAAGGGAAAAACGatgAAGAATAAGGCTtATAACAAAAAAAGGAACTTCCTGACCCATAGTGAAATCGAATCACTCCTTAAAGCAGCAAATACCGGGCCTCATGCAGCACGTAATaaaggagaagaacttttca	N/A	pBTRK_lacO_cmR_fimB_min_mut
amplification of pBAD-DsrA backbone for RseX insertion	GATCCCCGGGGATCCTCTAG	ATGGCCAAACAGTAGAGAGTTG	pBAD-RseX
amplification of RseX for assembly into pBAD-DsrA	ctctactgtttggccatTTTTTATTATTCTGTGTCATGATGC	ggatccccggggatcGATCAGGCGCACATTAATG	
SDM of pBAD-RseX for fimB assays	atgcttccgttataagcctgTTATCGTCTTGTTTATATTTTTTGG	catgacacagaataataactaATGGCCAAACAGTAGAGAG	pBAD-RseXmut-fimB
SDM of pBAD-RseX for ihfB assays	tgtgtgtctaGATGCTTCCGTTATTAGC	gtaataaaaaATGGCCAAACAGTAGAGAG	pBAD-RseXmut-ihfB

1.3 Supplementary Figures

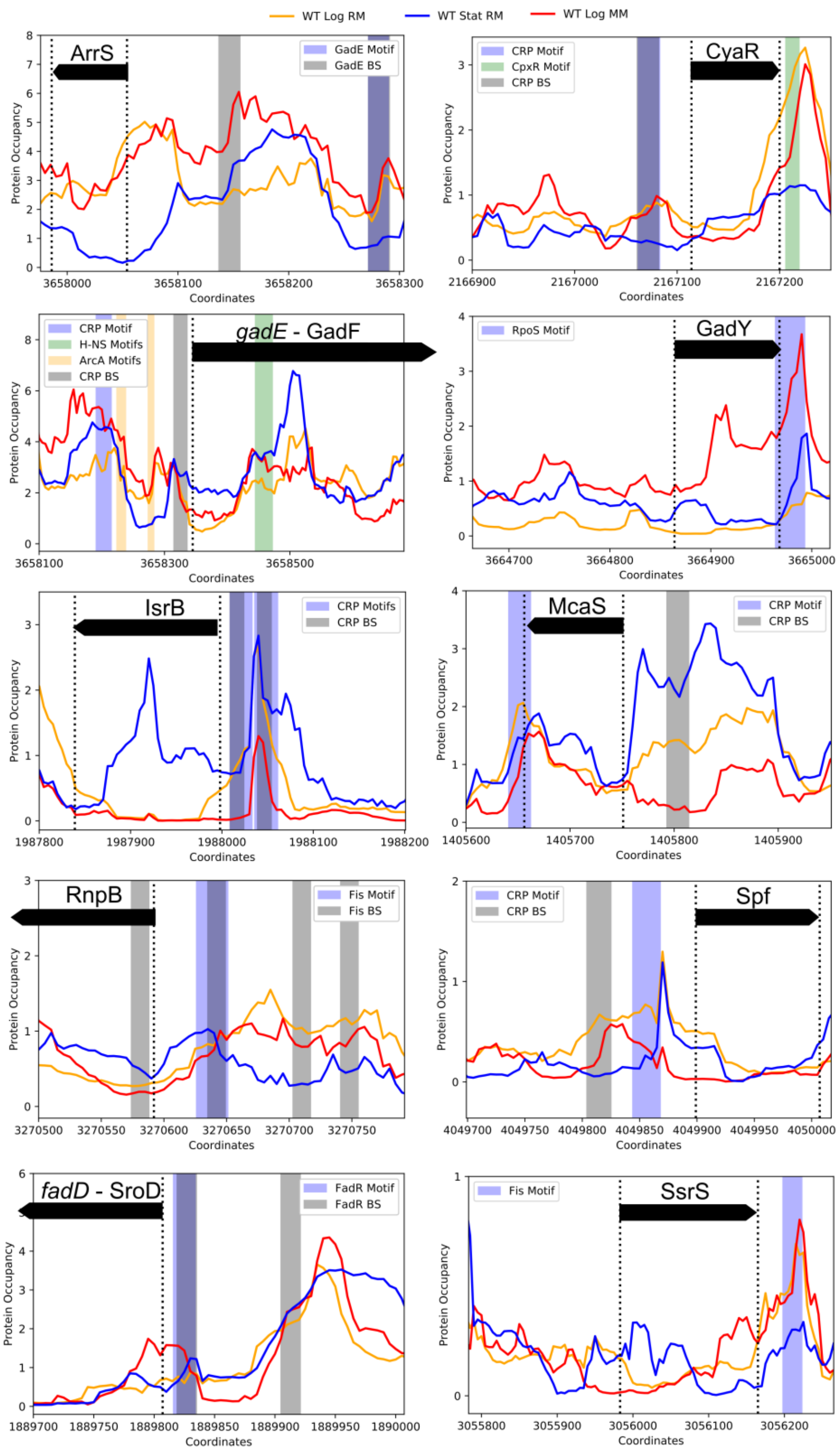

Supplementary Figure 1. IPOD-HR profiles of all captured positive controls. Known binding sites (grey) and predicted motifs (blue, green, or yellow) highlighted for each sRNA with documented DBP(s) captured in the high confidence set, with the exception of Fur-RyhB and PhoP-MgrR, which are showcased in Figure 2B/C.

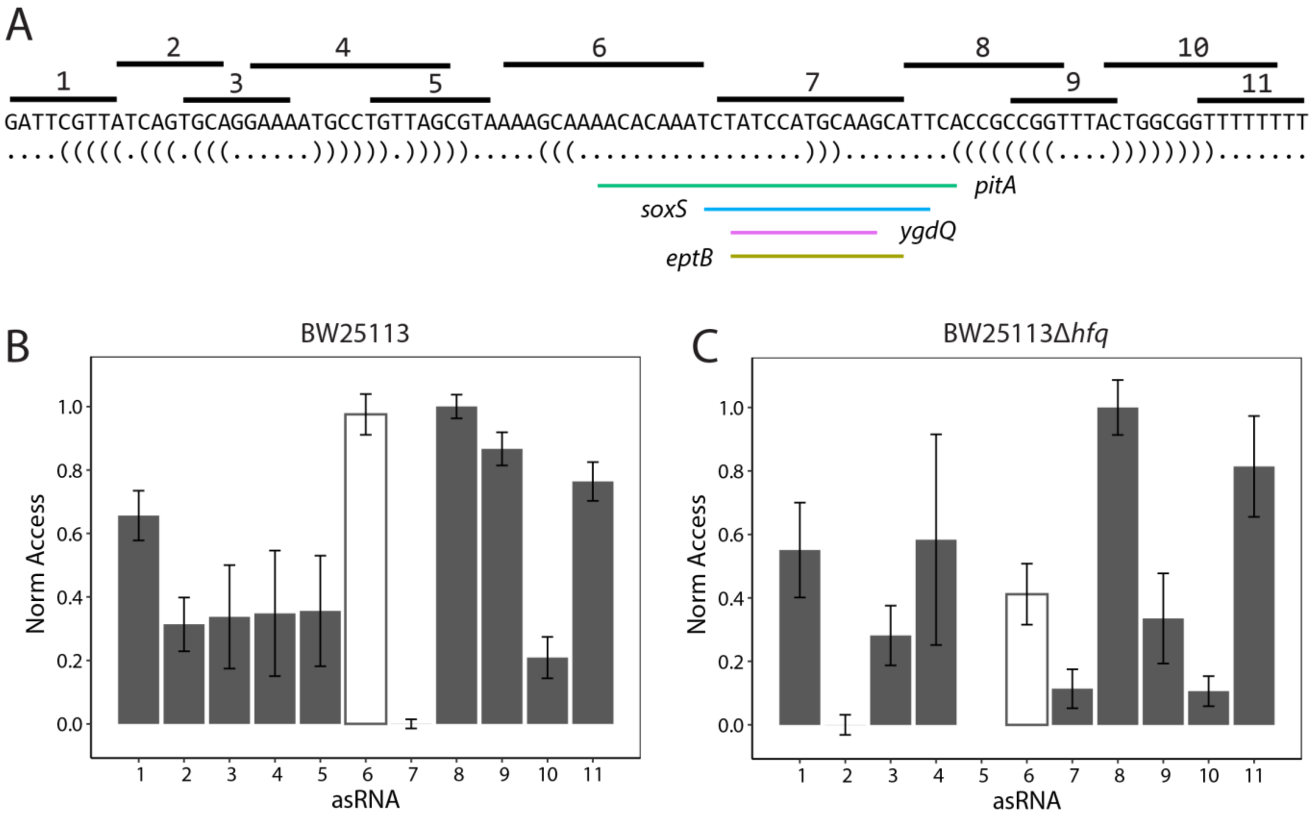

Supplementary Figure 2. Accessibility profiles of MgrR showcase functional region specificity. A. Target regions along MgrR. Binding sites corresponding to confirmed targets are indicated. B. Regional accessibility in BW25113. Regions 6 (nts 38-53) and 8 (nts 68-80) are selected as selected as likely-functional regions based on their distinctly high cognate-binding activity compared to neighboring regions. C. Regional accessibility profile of MgrR in the absence of cellular Hfq. Notably, the accessibility exhibits a net decrease, including that of proposed functional region 6, further supporting the regulatory potential of this region.

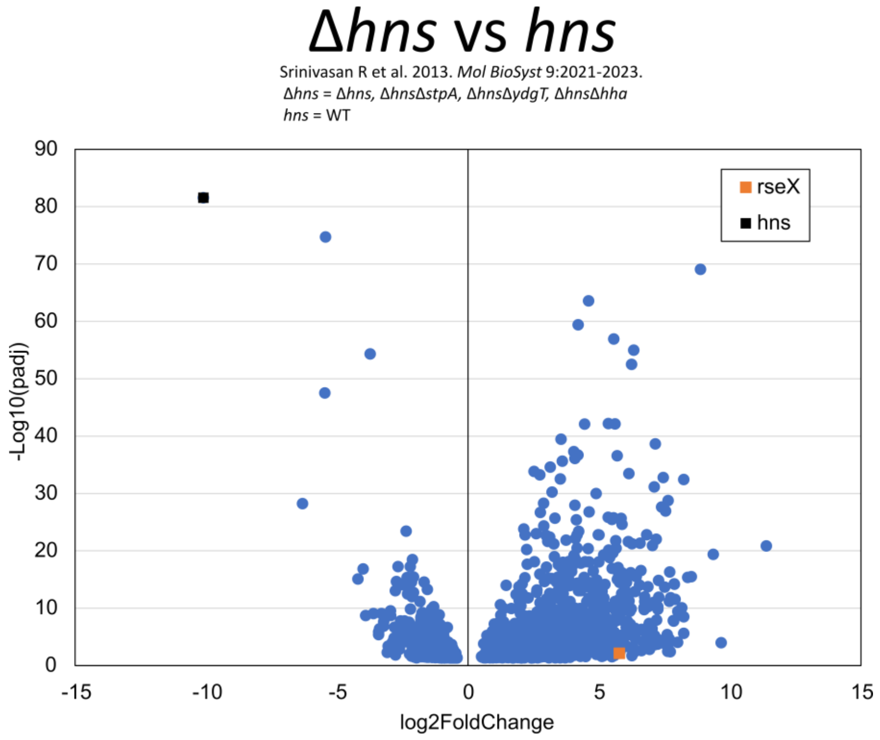

Supplementary Figure 3. Instances of significant differential expression under H-NS deletion highlight selective RseX induction. DESeq2 analysis for pooled *hns*-null vs *hns*-+ strains (Srinivasan et al., 2013) shows RseX (log2FC = 5.77 and padj = 7.24E-03) as the only envelope stress response-related sRNA to be significantly differentially expressed under H-NS deletion (MicA, MicC, MicF, RybB, OmrA, OmrB all did not show statistically significant differential expression, p-adj < 0.05).

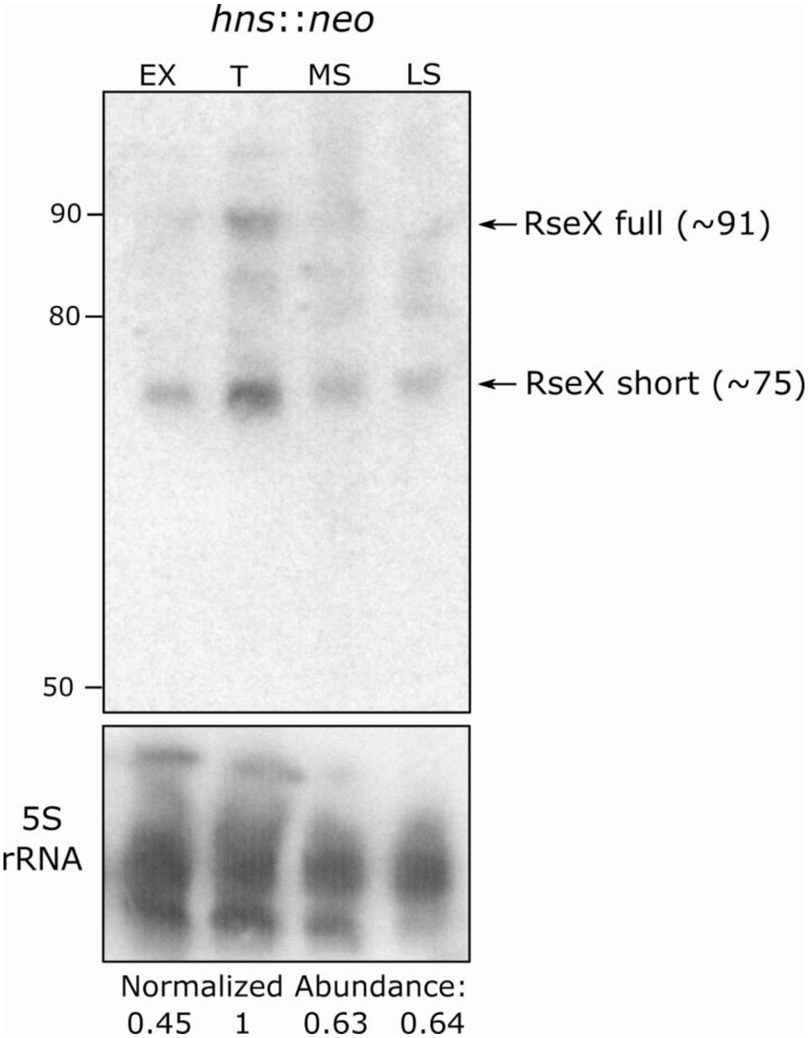

Supplementary Figure 4. Multiple native RseX transcripts detected by Northern blotting. To further corroborate the multiple transcripts seen in the northern blotting of an *hns* deletion, an additional northern was performed on an *hns::neo* mutant, which contains the N-terminal 37 amino acids suspected to allow some DNA binding activity. The two distinct transcript lengths were clearer compared to the *hns* deletion strain, and detected even in exponential phase (4 hours post-seeding) along with the previously detected transition to stationary (T, 7 hours post-seeding), mid stationary (MS, 24 hours post-seeding), and late stationary (LS, 48 hours post-seeding). Normalized abundance of the bottom, ~ 75 nt band to the 5S rRNA is similar between the 2 distinct *hns-null* strains (main text Fig. 5), with the exception of exponential growth phase.

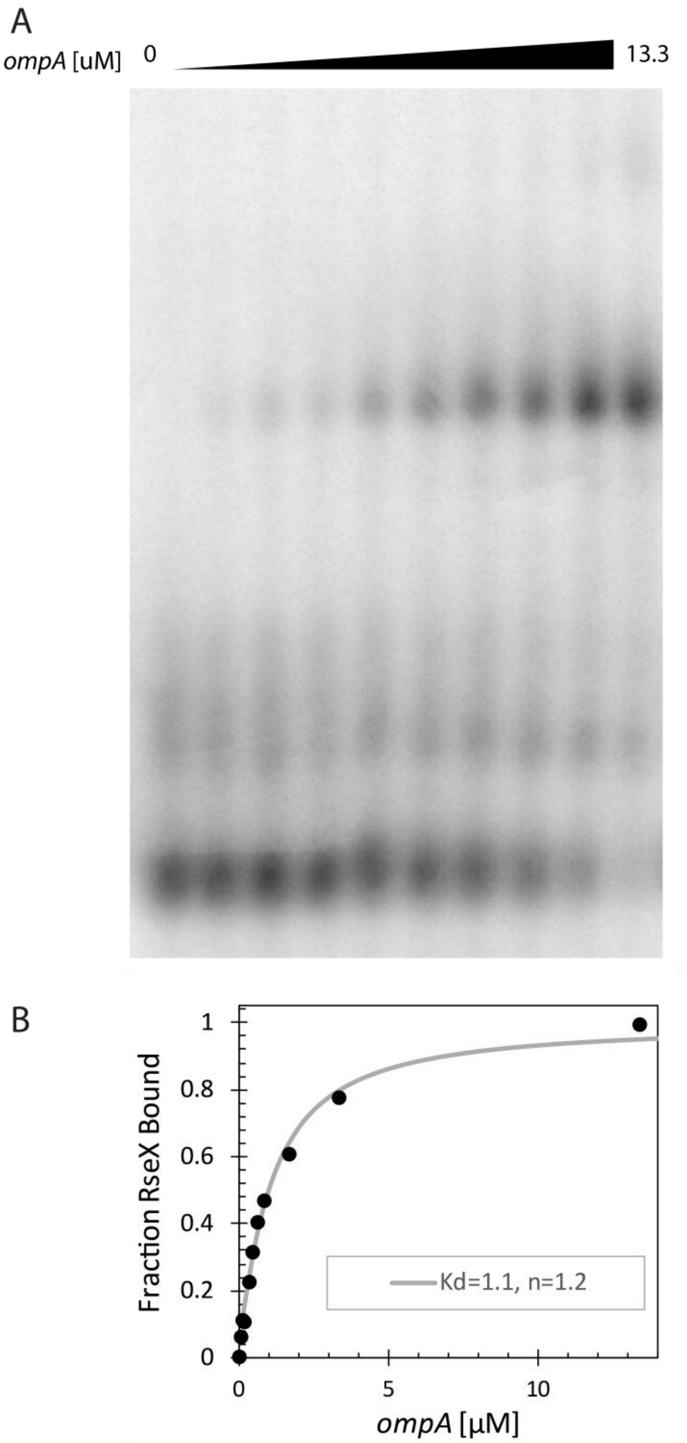

Supplementary Figure 5. *In vitro* binding assay between RseX and known target, *ompA*, suggests weaker interaction compared to *fimB* and *ihfB*. 1.3 pmol RseX was included in each binding reaction. Dissociation constants (K_D) for the RseX-*ompA* interaction was calculated as ~1.1 uM using the modified Hill equations (panel B) (Ryder et al., 2008). Notably, *ompA* has a higher K_D value than measured for the novel targets, *fimB* and *ihfB*, suggesting a weaker interaction.

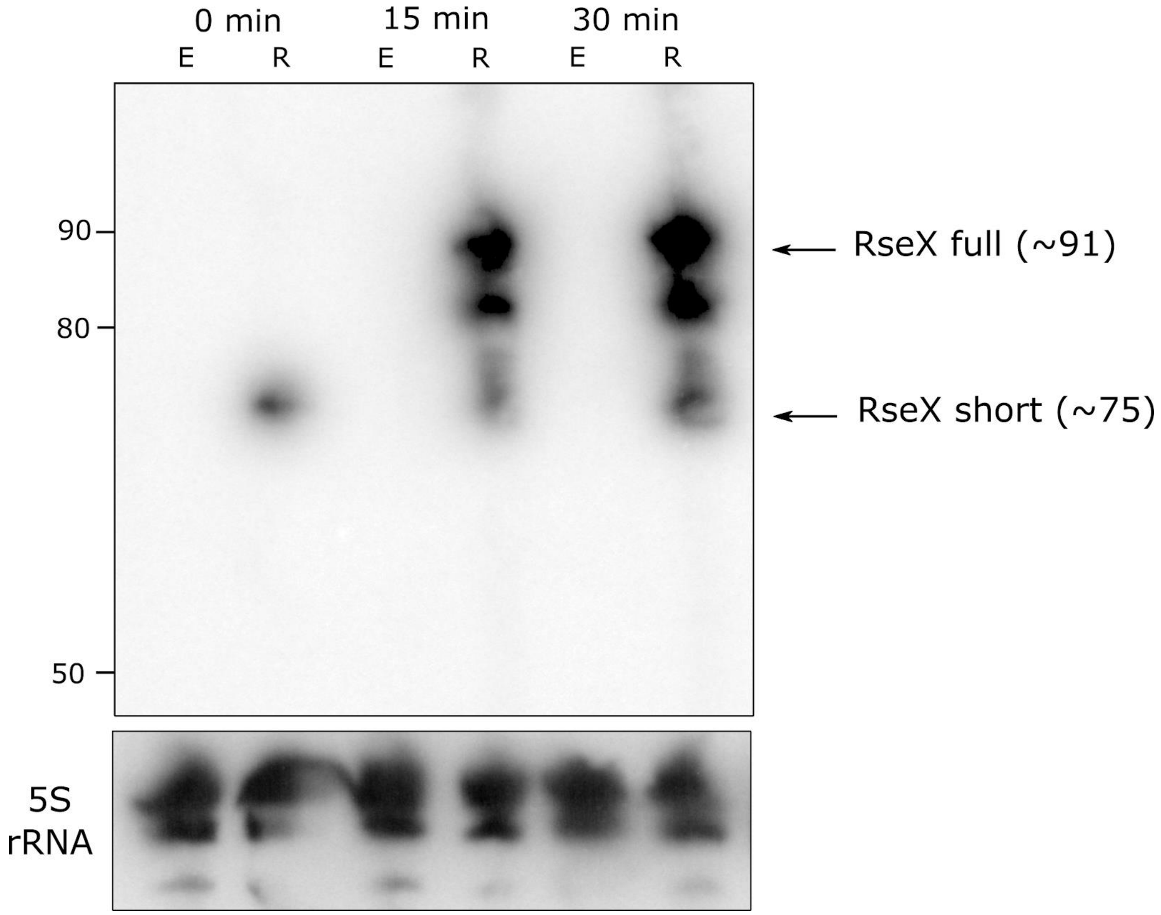

Supplementary Figure 6. High induction of RseX is achieved from a pBAD-RseX construct at 15 and 30 minutes post-induction with 0.05% arabinose. Expression of full RseX, an intermediate RseX product, and the short RseX product is observed following induction with 0.05% arabinose. Slight leaky expression of the short RseX product is observed prior to induction. As only the bottom band is observed under leaky conditions at 0 minutes, we propose that low levels of full RseX were amenable to full processing to the RseX short product. Note that top and middle RseX bands are saturated in the image at 15 and 30 minutes, suggesting extremely high expression compared to leakiness at 0 min (pre-induction).

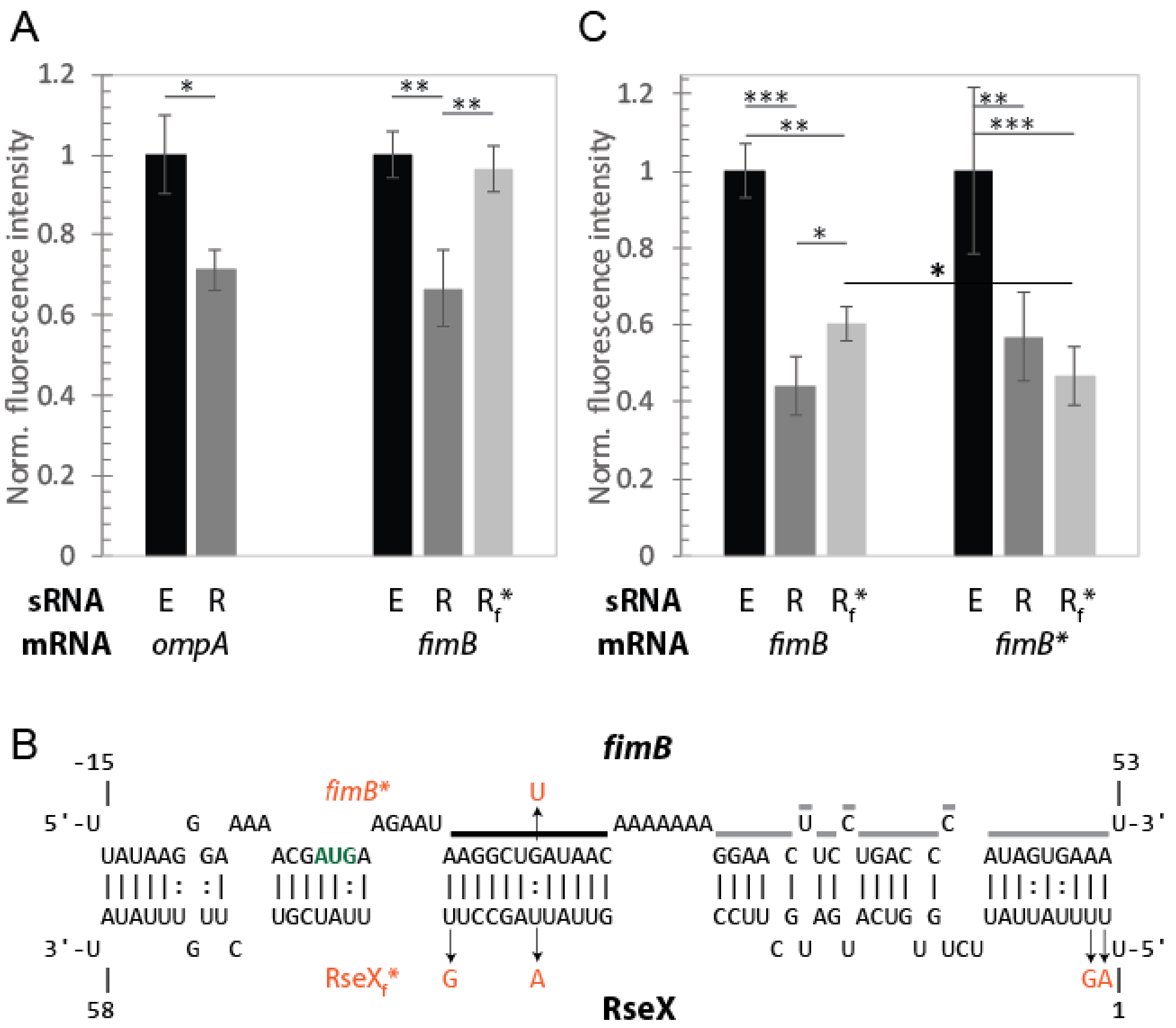

Supp. Fig. 7. Supplementary reporter assays for wildtype and mutant *fimB*. Illustrated means represent median fluorescence as normalized to respective pNM12 controls at OD_{600} 1; samples for each median were collected in at least triplicate. Error bars represent propagated standard deviation of the mean and asterisks indicate significant differences as evaluated by unpaired Student's t-test (p-value < 0.001, < 0.01, <0.05 are represented as ***, **, and *, respectively). A. Reporter assay shows full alleviation of RseX-*fimB* repression by the RseX$_f$* mutant (Fig. 9, main text) when induced with 0.01% arabinose (p-value < 0.01). B. A "minimal" *fimB* mutant, *fimB** (1 point mutation, outlined in orange), adhered to structure and codon design specifications described in Methods. C. When RseX/RseX$_f$* expression is induced with 0.05% arabinose, *fimB-gfp* repression by RseX is alleviated partially by 4 point mutations in RseX outlined in (B) (p-value < 0.05), as specified in main text Fig. 9. Notably, regulation by this RseX mutant is partially reconstituted in a minimal *fimB* mutant (*fimB**) (p-value < 0.05). However, the observed normalized fluorescence of the RseX$_f$*-fimB* construct is not significantly different from RseX (WT)-*fimB** regulation (p-value = 0.09).

Ryder, S.P., Recht, M.I., and Williamson, J.R. (2008). Quantitative analysis of protein-RNA interactions by gel mobility shift. *Methods Mol Biol* 488**,** 99-115. doi: 10.1007/978-1-60327-475-3_7.

Srinivasan, R., Chandraprakash, D., Krishnamurthi, R., Singh, P., Scolari, V.F., Krishna, S., et al. (2013). Genomic analysis reveals epistatic silencing of "expensive" genes in Escherichia coli K-12. *Mol Biosyst* 9(8)**,** 2021-2033. doi: 10.1039/c3mb70035f.
